# Gaussian process regression of HDR prostate biopsy dose for improved modelling of localization‐induced uncertainty

**DOI:** 10.1002/mp.70712

**Published:** 2026-11-01

**Authors:** Matthew Muscat, Juanita Crook, Andrew Jirasek, Jeffrey Andrews, Nathan Becker

**Affiliations:** ^1^ Department of Computer Science, Mathematics, Physics and Statistics, Irving K. Barber Faculty of Science University of British Columbia Okanagan Kelowna British Columbia Canada; ^2^ BC Cancer Kelowna British Columbia Canada; ^3^ Department of Radiation Oncology and Molecular Radiation Sciences Johns Hopkins University School of Medicine Baltimore Maryland USA; ^4^ Department of Surgery University of British Columbia Kelowna British Columbia Canada

**Keywords:** gaussian process regression, hdr brachytherapy, localization uncertainty, prostate biopsy dosimetry, probabilistic quality assurance

## Abstract

**Background:**

Biopsy‐level dose interpretation after high‐dose‐rate prostate brachytherapy is limited by localization uncertainty, and separate voxelwise Monte Carlo summaries do not explicitly account for the axial spatial correlation present along a biopsy core.

**Purpose:**

To develop and internally evaluate a biopsy‐level Gaussian process regression framework for assigning along‐core high‐dose‐rate prostate brachytherapy dose under localization uncertainty, and to test whether explicit modelling of axial spatial correlation improves uncertainty characterization relative to voxelwise Monte Carlo summaries treated independently.

**Methods:**

We analyzed 27 MR‐informed, transrectal‐ultrasound (TRUS)‐guided transperineal template biopsies from 15 patients treated with high‐dose‐rate prostate brachytherapy. For each biopsy voxel, Monte Carlo localization perturbations were propagated to generate dose summaries and voxel‐specific variances. Biopsy‐specific empirical semivariograms were fitted with candidate covariance kernels, and ordinary‐mean heteroscedastic Gaussian process posteriors were computed along each core. Internal evaluation included latent‐profile residual diagnostics, kernel‐sensitivity analysis, and blocked cross‐validation using contiguous held‐out axial segments scored on held‐out points only.

**Results:**

For the selected baseline kernel, Matérn ν=1.5, the across‐biopsy median voxel‐averaged standard deviation was 6.91 Gy for the Monte Carlo summaries and 2.60 Gy for the latent GP posterior. The median within‐biopsy reduction was 59.1%, with a median shrinkage ratio of 2.45. Fitted length scales were heterogeneous across biopsies, with median ${\hat{\ell }}_{b}=15.35$ mm, while nugget fractions were typically small. Cohort standardized residuals were near‐centered with conservative spread. In blocked cross‐validation, predictive performance on held‐out points remained favorable, with median root‐mean‐square error of 1.99 Gy, median mean absolute error of 1.58 Gy, and median negative log predictive density of 3.28. Among the tested kernels, held‐out predictive error favored Matérn ν=1.5.

**Conclusion:**

Along‐core dose interpretation after localization uncertainty should use a spatially correlated model rather than separate voxelwise summaries alone. This Gaussian process framework provides a covariance‐informed posterior dose representation with held‐out internal validation and offers a practical basis for biopsy‐level probabilistic quality assurance and future dose‐response analyses.

## 1 INTRODUCTION

High‐dose‐rate (HDR) brachytherapy is an established curative treatment for localized prostate cancer, with excellent long‐term biochemical control in appropriately selected patients.^1^ Yet local recurrence still occurs, and recurrence‐mapping studies indicate that failures can arise in or near sites of initial disease even when gross dosimetric coverage is high.^2^, ^3^, ^4^, ^5^ Imaging and pathology studies further show pronounced genomic, phenotypic, and microenvironmental heterogeneity within individual prostates and within single dominant lesions.^6^, ^7^, ^8^, ^9^ Together, these observations motivate biopsy‐scale dose assignment with explicit uncertainty quantification.

Multiparametric MRI and MRI‐directed biopsy pathways have improved localization of clinically significant disease and strengthened targeted sampling strategies.^10^, ^11^, ^12^, ^13^, ^14^ However, guidance does not make biopsy localization exact: MRI‐US fusion/registration uncertainty, contouring variability, needle delivery error, and prostate deformation remain relevant at millimetre scale.^15^, ^16^, ^17^, ^18^, ^19^, ^20^ In HDR brachytherapy, where dose can vary steeply over millimetres near the implanted source geometry, these geometric uncertainties can materially change the dose assigned to neighbouring locations along a core.

Post‐treatment biopsies can carry rich biochemical and molecular information, including Raman spectroscopy‐based signatures of radiation response and disease progression.^21^, ^22^ Interpreting these assays in a dose‐response framework requires spatially resolved dosimetry along each core‐needle biopsy, together with a credible uncertainty model. Here, a biopsy core refers to the thin strip of tissue extracted along the biopsy needle trajectory; for dose assignment, that geometry motivates treating each core as a one‐dimensional axial profile rather than a single representative point.

Previous work developed a Monte Carlo method that maps planned HDR dose onto millimetre‐scale voxels along MR‐informed, TRUS‐guided transperineal template core‐needle biopsies while propagating rigid localization uncertainty.^23^ For biopsy b, voxel v, and trial t, that work denoted mapped dose by $D_{b,v}^{\left(t\right)}$ (Gy), and reported voxel‐ and biopsy‐level summaries including dose‐volume histograms (DVHs) and distributions of local dose‐gradient magnitude ${∥\nabla D∥}_{b,v}^{\left(t\right)}=∥\nabla D^{\left(t\right)}∥$. By analysing finite‐difference dose increments along each core, that work showed that $\Delta D_{b}\left(h\right)$ saturates beyond a characteristic axial distance h, indicating axial coherence rather than voxel‐wise independence.^23^ In the cohort‐level analysis, mean absolute dose differences increased with axial separation before plateauing at approximately 1--1.5cm, with centimetre‐scale differences on the order of 10Gy, further supporting an along‐core covariance model rather than independent voxelwise summaries.^24^

The related cohort‐level analysis extended that framework to probabilistic biopsy‐level quality assurance (QA).^24^ In that study, biopsy‐level DVH metrics were treated as random variables under localization uncertainty, and pass probabilities for clinical DVH thresholds were estimated by Monte Carlo sampling. Biopsies were then assigned to QA categories, linking localization uncertainty to decision‐relevant cohort‐level outcomes. Quantitatively, comparing nominal voxel assignments with localization‐perturbed trial values gave a pooled mean absolute dose deviation of 7.6Gy, with a 95th percentile of 33.0Gy, emphasizing that millimetre‐scale localization uncertainty can translate into large biopsy‐scale dose‐assignment variability. Achieving 95% pass probability required sizeable nominal buffers (approximately 6Gy for $D_{98\%}$, 21Gy for $D_{50\%}$, 49Gy for $D_{2\%}$, and 49 percentage points for $V_{150\%}$).^24^

Related work used the same localization‐uncertainty Monte Carlo engine to derive along‐core probabilistic tissue‐class sampling profiles and spatially resolved DIL‐sampling descriptors, further supporting this framework as a common foundation for biopsy‐level QA and downstream translational analyses.^25^

In all of these earlier studies, the Monte Carlo engine was used in a purely non‐parametric manner: for each voxel (b,v), the distribution ${\left\{D_{b,v}^{\left(t\right)}\right\}}_{t}$ was summarised independently (e.g. empirical quantiles or moments), and downstream DVH calculations used these voxel‐wise samples without explicitly modeling along‐core spatial correlation. The coherence‐length analysis in Refs. 23, 24 made this correlation explicit, but did not exploit it to stabilise voxel‐wise dose estimates or their uncertainties. Thus, the open methodological gap was not whether localization uncertainty matters, but how to represent it in a form that is more stable for inference and QA interpretation. We address this by leveraging the underlying spatial covariance structure.

A spatial model is therefore needed. Independent voxelwise summaries are the simplest baseline, yet they ignore the axial coherence already evident in these data and therefore discard information shared by neighbouring voxels along the same core.^23^, ^24^ Conceptually, a core is a connected tissue sample, so neighbouring voxel summaries should not be treated as unrelated measurements when the underlying dose field varies continuously over local axial distances. Simpler non‐probabilistic smoothers such as kernel smoothing, spline smoothing, or LOWESS could regularise the mean along‐core profile, but they do not by themselves provide a biopsy‐specific covariance model with posterior uncertainty for downstream QA and dose‐response analyses. We therefore chose Gaussian process regression because it provides a flexible one‐dimensional spatial model with posterior mean and variance along the biopsy, accommodates voxel‐specific localization‐induced observation uncertainty, and links naturally to semivariogram‐based covariance estimation and kernel selection.^26^, ^27^, ^28^ This gives a statistically principled correlated model matched to the biopsy geometry, heteroscedastic Monte Carlo outputs, and validation framework used here.

In the present work, we use a Gaussian‐process regression (GPR) framework^26^, ^27^, ^29^ to model dose along each biopsy as a one‐dimensional spatial profile. We treat the unknown dose profile $D_{b}\left(z\right)$ (Gy) along axial coordinate z (mm) for biopsy b as a smooth latent function, and the Monte Carlo outputs as noisy observations of that function under the assumed localization‐uncertainty model. This lets nearby voxels along the core inform one another through spatial correlation, so the model can attribute smooth along‐core dose trends to the latent profile while representing voxel‐specific localization‐induced dose dispersion as heteroscedastic observation uncertainty. In turn, this yields less erratic uncertainty estimates than treating voxels independently.

The aims of this methodological paper are:

1. to estimate biopsy‐specific empirical semivariograms of Monte Carlo dose along each core, and fit Matérn models, a standard covariance family with tunable smoothness, to quantify fitted length scale and micro‐scale variance;
2. to construct biopsy‐specific GPR posteriors for $D_{b}\left(z\right)$ from per‐voxel Monte Carlo summaries with heteroscedastic noise, meaning voxel‐specific noise variances rather than one shared variance;
3. to quantify voxelwise and biopsy‐level uncertainty reduction relative to voxelwise Monte Carlo summaries treated independently; and
4. to summarize cohort‐level covariance and uncertainty‐reduction metrics for downstream dose‐response and QA interpretation.

Throughout, we treat the GPR model as a statistically principled smoother for the latent dose field along each core. We do not, in this paper, re‐compute biopsy‐level DVH pass probabilities; rather, we focus on formulating, fitting, and internally evaluating the correlated model and on quantifying how voxelwise posterior uncertainty changes when along‐core spatial structure is incorporated. Future work will integrate GPR‐based dose profiles into the probabilistic QA framework developed in a related cohort‐level analysis.

## 2 MATERIALS AND METHODS

Before detailing individual model components, we first provide a compact overview of the full analysis sequence (Figure 1). This schematic links the core steps (inputs, semivariogram‐based covariance fitting, and GP inference) to the validation branches (kernel sensitivity and blocked cross‐validation) and the final reported outputs.

**FIGURE 1:**
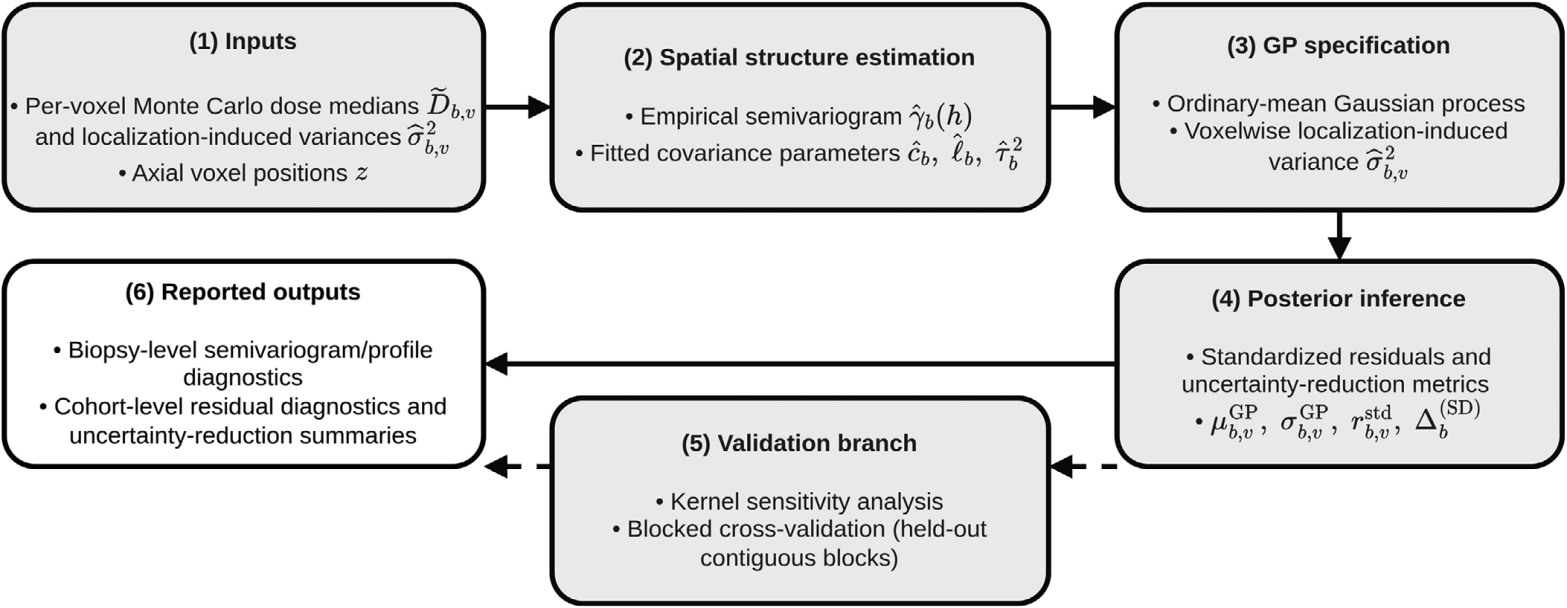
Overview of the biopsy‐level GPR methodology applied after Monte Carlo dose mapping: Monte Carlo voxel summaries are used to estimate biopsy‐specific semivariograms, fit covariance structure, infer GP posteriors, and produce posterior dose and uncertainty summaries evaluated through residual diagnostics, kernel‐sensitivity analysis, and blocked cross‐validation.

### 2.1 Clinical cohort and biopsy localisation

This study reuses the prospective HDR prostate brachytherapy cohort and biopsy‐localisation procedure described previously.^23^, ^24^, ^25^ Patients with localised prostate cancer in the HDR monotherapy arm received 27Gy in two fractions of 13.5Gy to the clinical target volume. We analyse only biopsies acquired during the second implantation procedure (after the first fraction and before the second fraction), so each analysed core corresponds to tissue exposed to one delivered fraction, typically about two weeks earlier.^23^, ^24^ In total, 27 MR‐informed, TRUS‐guided transperineal template core biopsies from 15 patients were available, targeting 26 mpMRI‐defined dominant intraprostatic lesions (DILs). The planning CT and MR images, HDR treatment plan, and biopsy trajectories were exported in DICOM format and processed using open‐source biopsy‐localisation, dosimetry, and statistical analysis software provided by BiopsyContext and BiopsyContext Dosimetry.^30^, ^31^

For each biopsy trajectory b, a one‐dimensional lattice of 1mm voxels was defined between the entry and exit points of the core inside the prostate, indexed by $v=1,\cdots ,n_{b}$ with axial coordinate $z_{b,v}$ (mm). The present analysis uses exactly the same set of biopsies and Monte Carlo realisations as in previous dosimetry and cohort‐level QA studies,^23^, ^24^ so that any differences in uncertainty quantification arise solely from the adoption of a correlated GPR model. The GPR analysis is applied after Monte Carlo dose mapping: it does not rerun the localization simulations, but models the resulting voxelwise dose summaries using an explicitly correlated along‐core representation.

### 2.2 Monte Carlo biopsy dosimetry and voxelwise summaries

Rigid localisation uncertainty was propagated using the Monte Carlo framework described in detail in Ref. 23. For each trial $t=1,\cdots ,N_{\mathrm{MC}}$ ($N_{\mathrm{MC}}=10,000$), an isotropic three‐dimensional Gaussian translation with σ=1.25mm was combined with an independent one‐sided axial offset sampled uniformly over any difference between the biopsy compartment and recovered tissue lengths. The resulting trial‐specific rigid displacement was applied to every voxel in the core. Dose at each perturbed voxel was obtained by inverse‐distance weighting over the four nearest dose‐lattice nodes, with weights proportional to reciprocal Euclidean distance, to obtain the trial‐specific dose

(1) $$D_{b,v}^{\left(t\right)}\;\left[\text{Gy}\right].$$

This is exemplified in Figure 2, which shows a single Monte Carlo trial for one biopsy, with the perturbed trajectory and the four nearest‐neighbour dose samples used to interpolate the dose at each perturbed voxel.

**FIGURE 2:**
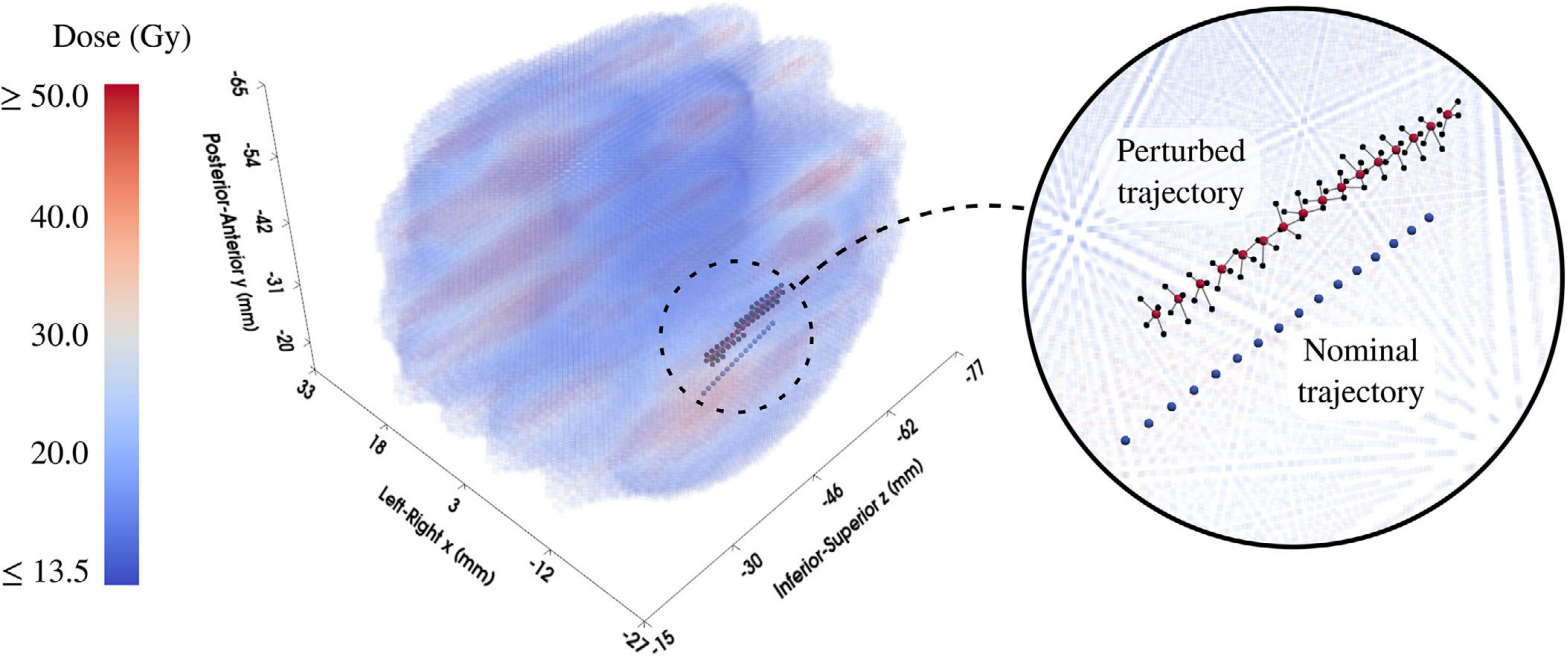
A rendering of the voxelised biopsy trajectory within the high‐dose‐rate brachytherapy dose field. The red points indicate the 1mm biopsy voxels for a single perturbed Monte Carlo realization, while the blue points indicate the nominal biopsy trajectory. The black points are the four nearest dose‐lattice samples used to interpolate the dose at each perturbed voxel, with connecting vectors indicating the corresponding interpolation neighbourhood. The HDR dose lattice is rendered in the background as a semi‐transparent volume. Dose‐lattice points below 13.5Gy have been omitted for clarity.

For each biopsy b and voxel v, the analyzed profile set comprised the nominal profile at t=0 and the $N_{\mathrm{MC}}=10\;000$ perturbed profiles, giving $T=N_{\mathrm{MC}}+1=10\;001$ profiles indexed by $t=0,\text{…},N_{\mathrm{MC}}$. This set was reduced to a target dose summary and an associated noise variance. We used the Monte Carlo median as the target,

(2) $${\tilde{D}}_{b,v}={\mathrm{median}}_{t}\;\left(D_{b,v}^{\left(t\right)}\right),$$

which is robust to occasional outlying trials. As a local measure of Monte Carlo dispersion, we computed the sample variance

(3) $${\hat{\sigma }}_{b,v}^{2}=\frac{1}{T-1}\sum_{t=0}^{N_{\mathrm{MC}}}{\left(D_{b,v}^{\left(t\right)}-{\bar{D}}_{b,v}\right)}^{2},$$

where ${\bar{D}}_{b,v}$ is the mean at (b,v) over the T analyzed profiles. We refer to ${\hat{\sigma }}_{b,v}=\sqrt{{\hat{\sigma }}_{b,v}^{2}}$ as the voxelwise Monte Carlo standard deviation. Importantly, ${\hat{\sigma }}_{b,v}^{2}$ quantifies localization‐induced dose dispersion under the assumed perturbation model and is used deliberately as a heteroscedastic observation‐uncertainty term; it is not the Monte Carlo sampling variance of the estimated median ${\tilde{D}}_{b,v}$. Equivalently, these voxelwise variances are the diagonal entries of the sample covariance matrix that would be obtained from the same trialwise dose vectors across the core; the present model retains these marginal variances but does not explicitly include the corresponding off‐diagonal cross‐voxel localization covariances.

For each biopsy, the axial coordinates together with the voxelwise targets and variances served as inputs to the semivariogram and GPR analyses.

### 2.3 Random‐field formulation and empirical semivariograms

For each biopsy, we use the Monte Carlo realisations to characterize spatial variation along a one‐dimensional dose field $D_{b}\left(z\right)$ and summarize axial correlation by an empirical semivariogram. For a stationary one‐dimensional process, the semivariogram^28^

(4) $$\gamma_{b}\left(h\right)=\frac{1}{2}\mathrm{Var}\;\left[D_{b}\left(z\right)-D_{b}\left(z+h\right)\right]$$

summarises how typical squared dose differences grow with axial separation h. Low values at short lags indicate that nearby voxel doses are similar; a rising semivariogram indicates decorrelation with axial distance; and a plateau, or sill, indicates that additional separation adds little further decorrelation. In the discrete voxelised setting, we estimated $\gamma_{b}\left(h\right)$ by averaging over all analyzed profiles and voxel pairs binned by physical axial separation in 1mm lag bins centered at $h_{k}=k\;mm$. A Matérn model was then fit to each biopsy‐specific semivariogram to estimate a length scale and nugget variance. The fitted Matérn length scale sets the axial distance scale of the structured covariance. For the baseline Matérn (ν=1.5) parameterisation used here, $h=\ell_{b}$ corresponds approximately to the half‐correlation distance: the structured latent‐dose correlation has fallen to $\left(1+\sqrt{3}\right)e^{-\sqrt{3}}\approx 0.48$ of its zero‐lag value. The partial sill is the spatially structured variance scale, while the nugget captures unresolved short‐scale variation or noise not explained by the smooth axial covariance model. Details of the semivariogram estimator, Matérn specification, and parameter interpretation are given in Supporting Information: Section S1.

### 2.4 Gaussian‐process regression of the dose profile

Conditioning on the per‐voxel Monte Carlo summaries, we model the latent dose profile $D_{b}\left(z\right)$ for each biopsy as a realisation of a stationary Gaussian process with a Matérn covariance function along the core, such that covariance depends on axial separation rather than absolute axial position^28^, ^32^ (Supporting Information: Section S1). In this framework the observed per‐voxel targets are treated as noisy samples of $D_{b}\left(z\right)$,

(5) $${\tilde{D}}_{b,v}=D_{b}\left(z_{b,v}\right)+\varepsilon_{b,v},$$

As a working approximation, although the shared rigid perturbations can induce cross‐voxel dependence, after conditioning on the spatially correlated latent dose profile the remaining voxelwise observation errors $\varepsilon_{b,v}$ are modelled as independent zero‐mean Gaussian terms with variance ${\hat{\sigma }}_{b,v}^{2}+{\hat{\tau }}_{b}^{2}$.^26^ Here ${\hat{\sigma }}_{b,v}^{2}$ is the Monte Carlo variance at the voxel and ${\hat{\tau }}_{b}^{2}$ is the fitted nugget variance for biopsy b from the semivariogram model.

For each biopsy we use the fitted Matérn hyperparameters $\left({\hat{c}}_{b},{\hat{\ell }}_{b},{\hat{\tau }}_{b}^{2}\right)$ and the training inputs $z_{b}$ and observations ${\tilde{D}}_{b}$ to compute the usual GPR posterior mean and variance of $D_{b}\left(z\right)$ along the core. In particular, evaluating the posterior at the training voxels yields, for each voxel v, a covariance‐informed dose estimate and residual uncertainty after borrowing information along the core,

(6) $$\mu_{b,v}^{\mathrm{GP}}=E\;\left[D_{b}\left(z_{b,v}\right)\;|\;{\tilde{D}}_{b}\right],\;\sigma_{b,v}^{\mathrm{GP}}=\sqrt{\mathrm{Var}\;\left[D_{b}\left(z_{b,v}\right)\;|\;{\tilde{D}}_{b}\right]}.$$

An ordinary‐mean (ordinary‐kriging) formulation was used, and posterior profiles were also evaluated on a finer axial grid for visualisation. Closed‐form expressions and covariance‐matrix details are provided in Supporting Information: Section S1.

The GPR analysis was implemented using the publicly available Python dosimetry software cited above. Empirical semivariograms were fitted by nonlinear least squares, and GP posterior means and variances were computed from fitted covariance matrices using Cholesky‐based linear solves.

### 2.5 Per‐biopsy uncertainty metrics

Using the voxelwise Monte Carlo standard deviations ${\hat{\sigma }}_{b,v}$ and the GP posterior standard deviations $\sigma_{b,v}^{\mathrm{GP}}$, we summarised uncertainty reduction and model fit for each biopsy b in several ways. As a basic voxelwise measure we defined the shrinkage ratio

(7) $$R_{b,v}=\frac{{\hat{\sigma }}_{b,v}}{\sigma_{b,v}^{\mathrm{GP}}},$$

and recorded, for each biopsy, the statistics of $R_{b,v}$ over voxels together with the fraction of voxels satisfying $R_{b,v}\geq 1.5$ (at least a 33.3% reduction in standard deviation).

As a biopsy‐level summary of voxelwise uncertainty, we defined

(8) $${\bar{\hat{\sigma }}}_{b}=\frac{1}{n_{b}}\sum_{v=1}^{n_{b}}{\hat{\sigma }}_{b,v},\;{\bar{\sigma }}_{b}^{\mathrm{GP}}=\frac{1}{n_{b}}\sum_{v=1}^{n_{b}}\sigma_{b,v}^{\mathrm{GP}},$$

and the corresponding biopsy‐level shrinkage ratio

(9) $${\bar{R}}_{b}=\frac{{\bar{\hat{\sigma }}}_{b}}{{\bar{\sigma }}_{b}^{\mathrm{GP}}}.$$

The percentage reduction in mean standard deviation was then

(10) $$\Delta_{b}^{\left(\mathrm{SD}\right)}=100\left(1-\frac{1}{{\bar{R}}_{b}}\right)\;.$$

This mean‐SD reduction is the quantity reported in per‐biopsy summaries and profile annotations.

For latent residual diagnostics, we summarized standardized residuals $r_{b,v}^{\mathrm{std},\mathrm{lat}}=\left({\tilde{D}}_{b,v}-\mu_{b,v}^{\mathrm{GP}}\right)/\sigma_{b,v}^{\mathrm{GP}}$ at biopsy level using $\mathrm{mean}(r_{b,v}^{\mathrm{std},\mathrm{lat}})$, $\mathrm{SD}(r_{b,v}^{\mathrm{std},\mathrm{lat}})$, and empirical coverage within ±1, ±2, and beyond ±3 standard deviations. MAE, RMSE, and NLPD are reported for blocked cross‐validation on held‐out voxels only (Section 2.7). From the semivariogram fits we carried forward, for each biopsy, the fitted length scale ${\hat{\ell }}_{b}$, partial sill ${\hat{c}}_{b}$, nugget variance ${\hat{\tau }}_{b}^{2}$, and a root‐mean‐square error between the empirical and fitted semivariograms as a basic measure of fit quality.

### 2.6 Cohort‐level summaries

Per‐biopsy metrics were aggregated across the cohort to obtain distributions of fitted length scales, nugget fractions, uncertainty reduction, and residual‐diagnostic summaries. We also examined the relation between voxelwise Monte Carlo and GPR uncertainties by regressing the per‐biopsy mean posterior standard deviation ${\bar{\sigma }}_{b}^{\mathrm{GP}}$ on the corresponding mean Monte Carlo standard deviation ${\bar{\hat{\sigma }}}_{b}$ using Deming regression assuming equal error variances for the two quantities.

### 2.7 Validation framework and baseline‐kernel selection

Model evaluation used latent residual diagnostics, kernel sensitivity, and blocked cross‐validation. Baseline and kernel‐sensitivity residuals were standardized by the latent GP posterior standard deviation and are interpreted as in‐sample diagnostics of the reconstructed latent profile. For blocked cross‐validation, primary held‐out residuals were standardized by $\sqrt{{\left(\sigma_{b,v}^{\mathrm{GP}}\right)}^{2}+{\hat{\sigma }}_{b,v}^{2}}$, which additionally includes the voxelwise localization‐induced dose dispersion; a latent‐only blocked‐CV definition was retained as a paired sensitivity analysis. We refer to these as the latent‐only and latent‐plus‐localization definitions, denoted by the superscripts lat and obs, respectively. The added variance in the latent‐plus‐localization definition is localization‐induced dose variance, not the sampling variance of the Monte Carlo median. Exact definitions are given in Supporting Information: Section S1. Summary statistics included $\mathrm{mean}(r_{b,v}^{\mathrm{std}})$, $\mathrm{SD}(r_{b,v}^{\mathrm{std}})$, and empirical coverage within ±1, ±2, and beyond ±3 standard deviations.

Kernel sensitivity analyses were run under a fixed modelling protocol (ordinary‐mean GPR, median target statistic, matched cohort and preprocessing), comparing four covariance choices spanning rough‐to‐smooth behaviour: exponential (Matérn ν=0.5), Matérn (ν=1.5), Matérn (ν=2.5), and squared‐exponential (RBF; limiting Matérn ν→∞) kernels. Kernel comparison was based on semivariogram fit quality, latent residual behaviour, and predictive performance under blocked cross‐validation. The baseline kernel used for primary reporting was Matérn (ν=1.5).

Blocked cross‐validation used contiguous axial hold‐outs with nominal target block length 8.0 mm, train‐only fitting, and held‐out‐only scoring. Partitions were adjusted when necessary to avoid very short held‐out segments, requiring each held‐out segment to contain at least 3 voxels and span at least 5.0 mm. At least two evaluable folds were retained per biopsy, with partitions rebalanced to two folds when required. Consequently, some realized held‐out segments exceeded 8.0 mm. Inference was computed per fold, then pooled by biopsy and summarized at cohort level. Predictive comparison metrics included held‐out MAE, RMSE, NLPD, and standardized‐residual summaries. This was the principal out‐of‐sample validation analysis and a key input to baseline‐kernel selection. The held‐out targets in this validation were Monte Carlo voxel summaries generated by the upstream localization and dosimetry model used in the companion dosimetry and QA analyses^23^, ^24^; blocked‐CV therefore evaluates internal prediction of held‐out Monte Carlo voxel‐summary targets, not agreement with an independently measured physical ground‐truth dose.

## 3 RESULTS

### 3.1 Representative biopsy‐level reconstructions

The two representative biopsies shown in Figures 3, 4 were selected to show distinct operating regimes of the same model. Biopsy 1 is an upper‐percentile uncertainty shrinkage example (biopsy‐level ratio ${\bar{R}}_{b}=2.88$, $\Delta_{b}^{\left(\mathrm{SD}\right)}=65.3\%$), while preserving close agreement between observed and reconstructed mean dose (${\bar{\tilde{D}}}_{b}=20.65$ Gy, ${\bar{\mu }}_{b}^{\mathrm{GP}}=20.53$ Gy). Biopsy 2 illustrates near‐zero mean bias with still substantial shrinkage (${\bar{R}}_{b}=2.58$, $\Delta_{b}^{\left(\mathrm{SD}\right)}=61.2\%$; ${\bar{\tilde{D}}}_{b}=26.48$ Gy, ${\bar{\mu }}_{b}^{\mathrm{GP}}=26.48$ Gy). Latent residual diagnostics for these two biopsies are consistent with the cohort pattern: Biopsy 1 $\mathrm{mean}(r_{b,v}^{\mathrm{std}})=0.055$, $\mathrm{SD}(r_{b,v}^{\mathrm{std}})=0.117$, and Biopsy 2 $\mathrm{mean}(r_{b,v}^{\mathrm{std}})=0.021$, $\mathrm{SD}(r_{b,v}^{\mathrm{std}})=0.146$. Additional biopsy‐level semivariograms and profile reconstructions are shown in Supporting Information: Section S2 (Figures S1, S2), to illustrate model behaviour beyond these two highlighted examples.

**FIGURE 3:**
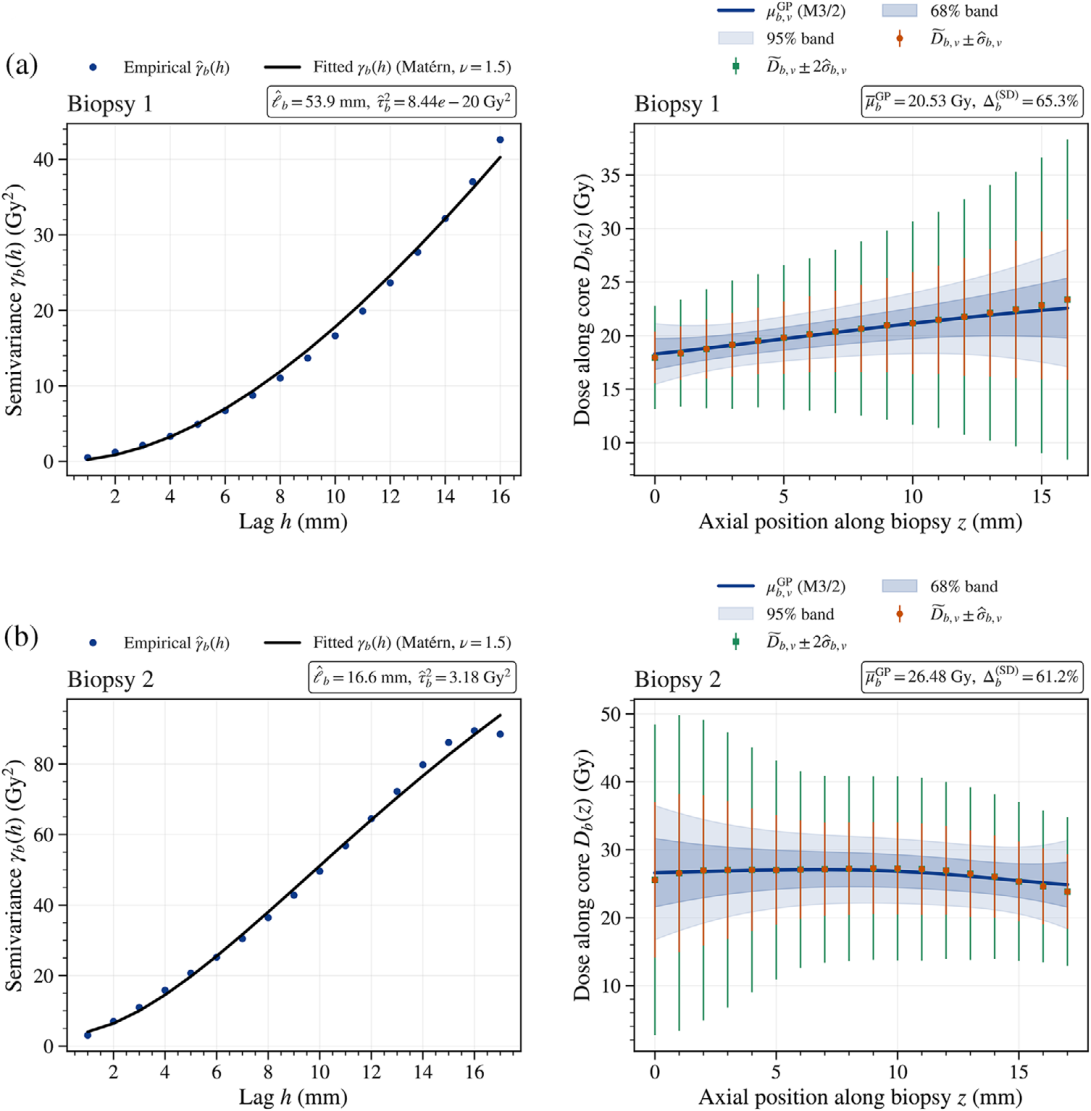
Representative examples illustrating the GPR covariance and dose reconstruction. Panels (a) and (b) show Biopsies 1 and 2, respectively. In each row, the left panel shows the empirical semivariogram ${\hat{\gamma }}_{b}\left(h\right)$ and the fitted Matérn model, with fitted length scale and nugget term indicated. The right panel shows the per‐voxel Monte Carlo medians ${\tilde{D}}_{b,v}$ and the GPR posterior mean profile $\mu_{b,v}^{\mathrm{GP}}$ with pointwise credible bands along the same biopsy.

**FIGURE 4:**
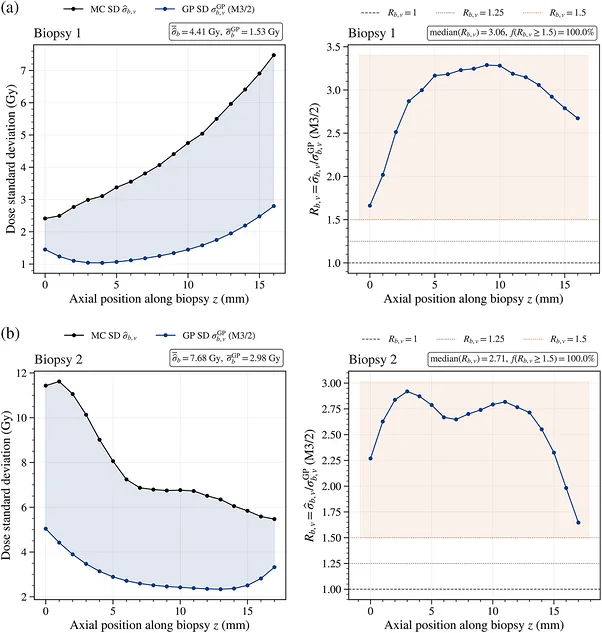
Representative examples displaying along‐core uncertainty reduction. Panels (a) and (b) show Biopsies 1 and 2, respectively. In each row, the left panel compares the voxelwise Monte Carlo standard deviation ${\hat{\sigma }}_{b,v}$ to the GPR posterior standard deviation $\sigma_{b,v}^{\mathrm{GP}}$ along the biopsy axis. The right panel shows the corresponding shrinkage ratio $R_{b,v}={\hat{\sigma }}_{b,v}/\sigma_{b,v}^{\mathrm{GP}}$, highlighting voxels with substantial reductions in uncertainty.

### 3.2 Cohort‐level covariance structure and uncertainty reduction

Across biopsies, the median voxel‐averaged Monte Carlo SD was 6.91Gy, and the median voxel‐averaged latent GP posterior SD was 2.60Gy. The median within‐biopsy reduction was 59.1%, with a median uncertainty ratio ${\bar{R}}_{b}=2.45$. At voxel level, the median biopsy had 95.5% of voxels with $R_{b,v}\geq 1.5$ (Table 1). As shown in Figure 5, the fitted length scales are heterogeneous across biopsies (median ${\hat{\ell }}_{b}=15.35$ mm; IQR 6.22--47.50 mm), while nugget fractions remain concentrated near zero with only a small upper tail (median ${\hat{\tau }}_{b}^{2}/\left({\hat{c}}_{b}+{\hat{\tau }}_{b}^{2}\right)$ ≈0; $Q_{75}=0.007$, $Q_{95}=0.019$); this indicates that nugget variance was generally small relative to the fitted spatially correlated variance. As shown in Figure 6, proportional uncertainty reduction is broadly stable across uncertainty scales (Bland‐Altman), while absolute SD reduction increases with scale (Deming slope =0.43, $R^{2}=0.92$). Cohort distributions of semivariogram fit RMSE and biopsy‐level shrinkage ratio are provided in Supporting Information: Figure S3.

**TABLE 1:** Cohort summary of the biopsy‐level metrics ${\bar{\hat{\sigma }}}_{b}$, ${\bar{\sigma }}_{b}^{\mathrm{GP}}$, ${\bar{R}}_{b}$, $\Delta_{b}^{\left(\mathrm{SD}\right)}$, and $\mathrm{Pct}(R_{b,v}\geq 1.5)$ defined in eqns. (7), (8), (9), (10) over 27 biopsies.

	**Median (IQR)**	5^th^–95^th^ percentile
${\bar{\hat{\sigma }}}_{b}\;\left[\text{Gy}\right]$	6.91(11.76)	0.85–33.12
${\bar{\sigma }}_{b}^{\mathrm{GP}}\;\left[\text{Gy}\right]$	2.60(5.94)	0.31–11.36
$\Delta_{b}^{\left(\mathrm{SD}\right)}\;\left[\%\right]$	59.1(6.8)	48.0–66.5
${\bar{R}}_{b}$	2.45(0.41)	1.92–2.99
$\mathrm{Pct}\left(R_{b,v}\geq 1.5\right)\;\left[\%\right]$	95.5(7.4)	55.8–100.0

**FIGURE 5:**
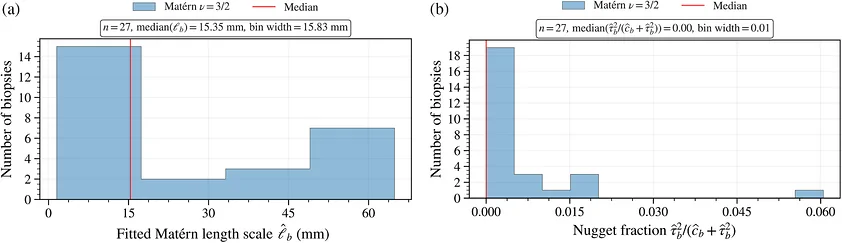
Cohort‐level summaries of the fitted Matérn covariance structure. Panel (a) shows the distribution of fitted length scales along the biopsies. Panel (b) shows the fitted nugget fraction ${\hat{\tau }}_{b}^{2}/\left({\hat{c}}_{b}+{\hat{\tau }}_{b}^{2}\right)$, representing nugget variance relative to the sum of nugget and spatially correlated variance.

**FIGURE 6:**
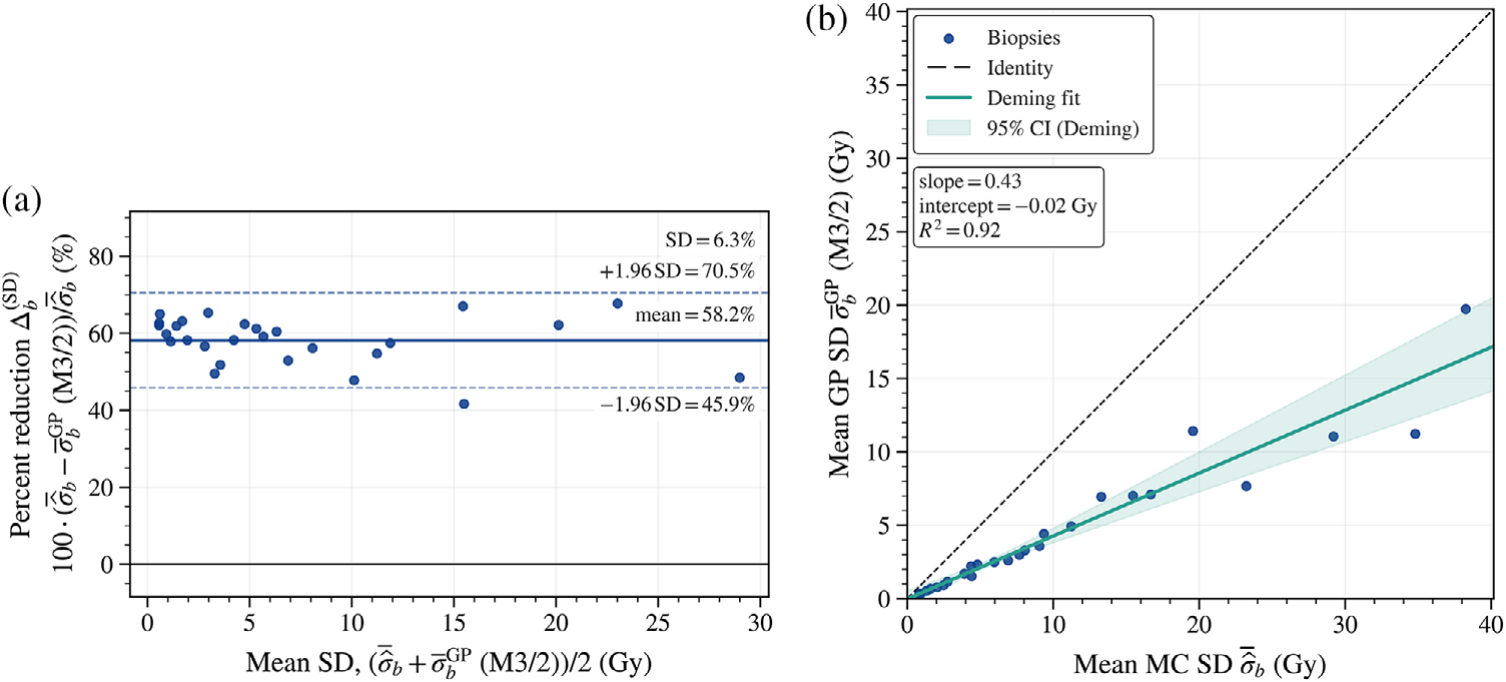
Cohort‐level uncertainty reduction. Panel (a) shows a Bland‐Altman plot of percent SD reduction $\Delta_{b}^{\left(\mathrm{SD}\right)}$; the solid and dashed lines indicate the mean and 95% limits of agreement. Panel (b) shows mean GPR posterior standard deviation versus mean voxelwise Monte Carlo standard deviation, with Deming regression (slope=0.43, $R^{2}=0.92$). A slope below 1 indicates larger absolute SD reduction at higher uncertainty scale.

### 3.3 Latent residual diagnostics for the baseline Matérn model

Latent residual behaviour for the baseline Matérn (ν=1.5) model was assessed using biopsy‐level summaries of $\mathrm{mean}(r_{b,v}^{\mathrm{std}})$, $\mathrm{SD}(r_{b,v}^{\mathrm{std}})$, and empirical coverage metrics. Detailed per‐biopsy and cohort‐level residual diagnostics are provided in Supporting Information: Section S3 (Figures S4–S6 and Table S1). Across biopsies, the median $\mathrm{mean}(r_{b,v}^{\mathrm{std}})$ was 0.052(0.025--0.079), the median $\mathrm{SD}(r_{b,v}^{\mathrm{std}})$ was 0.157(0.107--0.310), and the median biopsy‐level empirical coverage was 100% within both ±1 and ±2 standard deviations. For the representative biopsies shown in Figures 3, 4, this behaviour is similar (Biopsy 1: $\mathrm{mean}(r_{b,v}^{\mathrm{std}})=0.055$, $\mathrm{SD}(r_{b,v}^{\mathrm{std}})=0.117$; Biopsy 2: $\mathrm{mean}(r_{b,v}^{\mathrm{std}})=0.021$, $\mathrm{SD}(r_{b,v}^{\mathrm{std}})=0.146$); see Supporting Information: Figures S4 and S5, for biopsy‐level residual traces and histograms.

### 3.4 Kernel sensitivity and baseline‐kernel choice

Kernel sensitivity analyses showed that the main conclusions regarding spatial coherence and uncertainty reduction were stable across kernel families, with modest differences in predictive and residual‐diagnostic performance (table 2).

**TABLE 2:** Compact kernel‐comparison summary using median $(Q_{25}$–$Q_{75})$ over biopsies. The first row reports within‐biopsy semivariogram‐fit RMSE; remaining rows report blocked‐CV held‐out predictive and residual‐diagnostic metrics under contiguous spatial hold‐outs.

Metric	Exp	Matérn ν=1.5	Matérn ν=2.5	RBF
Semivar. RMSE	5.58 (1.68–24.05)	1.45 (0.36–7.69)	1.11 (0.33–8.48)	1.39 (0.31–8.98)
Blocked‐CV RMSE (Gy)	2.29 (1.18–3.75)	1.99 (1.13–3.36)	2.15 (1.12–3.40)	2.33 (1.12–3.51)
Blocked‐CV MAE (Gy)	1.99 (0.96–3.16)	1.58 (0.80–2.98)	1.67 (0.85–3.03)	1.81 (0.87–3.04)
Blocked‐CV NLPD	3.26 (2.59–4.14)	3.28 (2.53–4.16)	3.27 (2.52–4.15)	3.26 (2.51–4.13)
$\mathrm{mean}(r_{b,v}^{\mathrm{std},\mathrm{obs}})$	−0.006 (‐0.029–0.071)	−0.016 (‐0.040–0.105)	−0.014 (‐0.039–0.119)	−0.010 (‐0.037–0.113)
$\mathrm{SD}(r_{b,v}^{\mathrm{std},\mathrm{obs}})$	0.214 (0.101–0.465)	0.220 (0.091–0.358)	0.203 (0.101–0.358)	0.212 (0.104–0.363)

The baseline kernel was selected as Matérn (ν=1.5) from the combined evidence in table 2: best blocked‐CV RMSE/MAE, residual‐diagnostic behaviour comparable to alternative kernels, and semivariogram‐fit quality that remained within the same order as the best‐performing smooth alternatives. Matérn (ν=2.5) achieved slightly lower semivariogram RMSE but did not improve held‐out prediction performance for the Monte Carlo voxel‐summary target relative to Matérn (ν=1.5). Operationally, kernel selection was therefore driven by held‐out prediction error for this target first (RMSE/MAE), with residual behaviour used as a consistency check. Full kernel‐stratified diagnostics are provided in Supporting Information: Section S4 (Figures S7–S10).

### 3.5 Blocked cross‐validation

Blocked cross‐validation used contiguous spatial hold‐outs with scoring restricted to held‐out voxels only. For the selected Matérn (ν=1.5) model, cohort‐level biopsy medians were RMSE=1.99 Gy (1.13--3.36), MAE=1.58 Gy (0.80--2.98), and NLPD=3.28(2.53--4.16), with $\mathrm{mean}(r_{b,v}^{\mathrm{std},\mathrm{obs}})=-0.016$ and $\mathrm{SD}(r_{b,v}^{\mathrm{std},\mathrm{obs}})=0.220$ on held‐out points; this is consistent with a near‐zero standardized‐residual mean and a conservative standardized‐residual spread under held‐out evaluation. Biopsy‐level median threshold coverage was non‐discriminative across kernels (100% within $|r_{b,v}^{\mathrm{std},\mathrm{obs}}|\leq 1$ and $|r_{b,v}^{\mathrm{std},\mathrm{obs}}|\leq 2$, and 0% for $|r_{b,v}^{\mathrm{std},\mathrm{obs}}|\geq 3$). These blocked‐CV results were used directly in baseline‐kernel selection. Cohort blocked‐CV summaries are reported in table 2, and expanded kernel‐stratified held‐out metrics, residual diagnostics, and representative fold‐level examples are provided in Supporting Information: Section S5 (Figures S11, S12 and Table S2). Variance‐mode sensitivity showed consistent conservatism for the latent‐plus‐localization definition relative to the latent‐only definition: $\Delta \mathrm{SD}\left(r_{b,v}^{\mathrm{std}}\right)=\mathrm{SD}\left(r_{b,v}^{\mathrm{std},\mathrm{obs}}\right)-\mathrm{SD}\left(r_{b,v}^{\mathrm{std},\mathrm{lat}}\right)$ was negative across kernels (about −0.13 to −0.09), while $\Delta \mathrm{mean}\left(r_{b,v}^{\mathrm{std}}\right)=\mathrm{mean}\left(r_{b,v}^{\mathrm{std},\mathrm{obs}}\right)-\mathrm{mean}\left(r_{b,v}^{\mathrm{std},\mathrm{lat}}\right)$ remained close to zero and ΔNLPD was positive (∼0.26 to 0.31); see Supporting Information: Table S2.

## 4 DISCUSSION

### 4.1 Principal findings

The present analysis supports a spatially correlated interpretation of along‐core dose after localization uncertainty propagation. Relative to voxelwise Monte Carlo summaries treated independently, the baseline Matérn (ν=1.5) model achieved substantial cohort‐level uncertainty reduction, while preserving near‐zero standardized‐residual means and subunit standardized‐residual spread. Quantitatively, across biopsies, the median voxel‐averaged Monte Carlo SD was 6.91 Gy and the median voxel‐averaged latent GP posterior SD was 2.60 Gy. The median within‐biopsy reduction was $\Delta_{b}^{\left(\mathrm{SD}\right)}=59.1\%$ (median ${\bar{R}}_{b}=2.45$). Under contiguous blocked‐CV hold‐outs, held‐out prediction performance remained favorable for the Monte Carlo voxel‐summary target (median RMSE =1.99 Gy, MAE =1.58 Gy), showing that the fitted spatial model retains predictive utility beyond the in‐sample reconstruction. Notably, this benefit was not restricted to low‐uncertainty biopsies. Across the cohort, proportional uncertainty reduction remained broadly stable, while the absolute reduction in SD increased with baseline uncertainty, indicating that the largest absolute gains occurred in biopsies with the greatest initial voxelwise Monte Carlo uncertainty.

### 4.2 Interpretation and model‐choice implications

The central methodological point is that axial covariance should be modeled explicitly when assigning dose context along biopsy cores. Independent voxelwise summaries discard coherence information that is present in these data and visible in empirical semivariograms. Kernel sensitivity and blocked‐CV together show that kernel selection should be driven primarily by held‐out predictive error, with residual behaviour as a consistency criterion. In this cohort, that combined criterion favored Matérn (ν=1.5): semivariogram‐fit quality remained competitive and blocked‐CV RMSE/MAE were best among tested kernels. Importantly, the observed uncertainty contraction is a covariance‐informed reduction in posterior uncertainty conditional on the upstream localization model used in those companion analyses^23^, ^24^, not a removal of the underlying localization uncertainty itself. It leverages the physical constraint that each biopsy is a connected tissue sample along one core trajectory, so neighboring voxels can constrain one another through the fitted covariance instead of being treated as independent.

This also clarifies why we used GPR rather than a simpler smoother alone. The objective was not merely to produce a visually smooth along‐core dose curve, but to obtain a covariance‐informed posterior reconstruction with uncertainty that could be quantified voxelwise, evaluated under blocked cross‐validation, and propagated into downstream probabilistic analyses. In that sense, kernel smoothing, spline smoothing, or LOWESS are useful descriptive baselines, but they are less well matched to the present objective because the key estimand is the paired posterior summary $\left(\mu_{b,v}^{GP},\sigma_{b,v}^{GP}\right)$, not just a smoothed mean profile, under a biopsy‐specific spatial covariance model with heteroscedastic noise.^26^, ^28^ GPR is therefore a natural correlated model for this task.

### 4.3 Translational relevance

In practical terms, correlated GP reconstruction converts independent voxelwise Monte Carlo summaries into a biopsy‐specific posterior dose representation along each core. This is directly relevant to downstream analyses that require voxel‐ or segment‐level dose context (e.g., biochemical, Raman, or genomic correlates), and it provides a cleaner statistical interface for the biopsy‐level probabilistic QA framework developed in related studies. ^23^, ^24^, ^25^ The key translational output is the paired posterior summary $\left(\mu_{b,v}^{\mathrm{GP}},\sigma_{b,v}^{\mathrm{GP}}\right)$: the reconstructed mean supports spatial dose attribution, while $\sigma_{b,v}^{\mathrm{GP}}$ provides a covariance‐informed residual uncertainty quantity. Together with the fitted covariance, this posterior representation can be sampled or propagated into probabilistic thresholding, weighted downstream analyses, or future dose‐response models. Because held‐out predictive error is quantified by blocked‐CV, this same framework can be extended to mapped‐core analyses (e.g., biopsy‐specific affine offset/scale models) with explicitly reported out‐of‐sample uncertainty behavior. The present study does not test whether this representation improves patient‐outcome prediction; rather, it supplies the dose‐attribution and uncertainty‐propagation representation needed for such analyses. In this cohort, that interface combines substantial uncertainty contraction (median $\Delta_{b}^{\left(\mathrm{SD}\right)}\approx 59\%$) with favorable held‐out performance (blocked‐CV median RMSE =1.99 Gy), supporting its use as a model‐conditional posterior dose representation before QA or dose‐response modeling.

### 4.4 Limitations

Several limitations should be stated explicitly. First, localization uncertainty propagation is currently rigid‐shift based and does not yet include nonrigid deformation. Because each Monte Carlo trial applies a common rigid perturbation to the complete biopsy core, localization‐induced dose perturbations are generally correlated across voxels. The current observation model retains the voxelwise localization‐induced variances but not these cross‐voxel error covariances; dependent Gaussian observation noise can in general be represented through a structured noise covariance.^26^ Within the working model, spatial correlation in the latent dose profile is represented separately through the full Matérn covariance, and the effect of the observation‐error approximation was not evaluated separately. Posterior variances are conditional on the fitted semivariogram hyperparameters and do not propagate uncertainty in their estimation. The stationary formulation does not represent position‐dependent covariance along the core. Because available lag support is limited by core length, fitted Matérn length scales approaching or exceeding the observed core span are only partially constrained by the data. Such values indicate persistent correlation across the sampled core but should not be interpreted as precise estimates of the distance at which decorrelation would occur beyond the observed domain. Second, this is a single‐cohort analysis with internal blocked‐CV validation and no external cohort. The blocked‐CV results therefore support internal predictive consistency for held‐out Monte Carlo voxel summaries, but should not be interpreted as validation against independently measured delivered dose. Third, the upstream dose lattice comes from the previously described biopsy‐dosimetry analysis^23^, ^24^ and was not recomputed with model‐based heterogeneous‐medium transport in this work. Finally, this paper is methodological and does not yet re‐estimate biopsy‐level DVH pass probabilities under GP posterior sampling.

### 4.5 Future directions

Future statistical work could retain pair‐specific semivariances, averaged over Monte Carlo profiles within each voxel pair, to assess whether semivariance at a common separation varies systematically with pair location along the core and thereby directly evaluate the stationarity assumption. It could also develop models that retain cross‐voxel localization‐induced covariance arising from shared rigid perturbations while separating this component from the latent spatial covariance represented by the GP. Such separation would be necessary to avoid double‐counting spatial structure when both covariance components are informed by the same localization‐perturbed profile ensemble.

Additional translational and validation extensions include four directions. First, biopsy‐specific mapping uncertainty could be modeled explicitly to help reconcile tissue‐length mismatch between the planned biopsy geometry and the recovered specimen coordinates, for example by allowing biopsy‐specific axial offset/scale or monotone warping with uncertainty propagated through the GP model.^23^, ^24^, ^25^ Second, GP posterior sampling could be integrated into the existing probabilistic QA framework to compare covariance‐informed pass probabilities against direct Monte Carlo estimates.^24^ Third, the same analysis could be repeated using heterogeneous‐medium brachytherapy dose calculations, such as TG‐186‐aligned Monte Carlo methods, rather than only the current TG‐43‐style water‐medium calculation.^33^, ^34^, ^35^ Fourth, external physical calibration would require a prospective phantom study with an independently measured dose reference. A staged validation pathway described in the companion along‐core dosimetry study separates sensitivity to the assumed localization prior from end‐to‐end physical calibration and proposes repeated biopsy‐compatible gel‐dosimetry experiments that reproduce the clinical imaging, localization, reconstruction, and specimen‐recovery workflow.^23^ For the present GPR framework, measured whole‐core or segment‐level doses could be compared with the corresponding posterior predictive distributions by quantifying systematic differences between measured and predicted doses and the proportion of measurements that fall within prespecified posterior prediction intervals. Such a study would distinguish external physical calibration of the localization and dose‐assignment workflow from the internal held‐out prediction of Monte Carlo‐derived voxel targets reported here.

## 5 CONCLUSION

We developed and internally evaluated a biopsy‐level Gaussian process framework for interpreting along‐core HDR prostate dose under localization uncertainty. By fitting biopsy‐specific semivariograms and using ordinary‐mean GPR with heteroscedastic voxelwise noise, the method preserved dose‐profile structure while substantially reducing apparent voxelwise uncertainty relative to separate voxelwise Monte Carlo summaries. Kernel sensitivity and blocked cross‐validation supported Matérn (ν=1.5) as the baseline choice in this cohort. These results support correlated random‐field modeling as a principled statistical method for along‐core dose interpretation and provide a model‐conditional posterior dose representation for future biopsy‐level probabilistic QA and dose‐response analysis.

## FUNDING INFORMATION

This research was supported by the Natural Sciences and Engineering Research Council of Canada (RGPIN‐2020‐07232, RGPIN‐2020‐04646), the University of British Columbia Okanagan, institutional research funds from BC Cancer, the BC Cancer Foundation (Kelowna Division), the Motorcycle Ride for Dad, and the Walter C. Sumner Fellowship.

## CONFLICT OF INTEREST STATEMENT

The authors declare no conflicts of interest.

## Supporting information

Supporting Information

## Data Availability

The imaging data used in this study consist of clinical DICOM datasets from patients treated at BC Cancer that were de‐identified and processed using the methods described above. The original DICOM files and derived patient‐level datasets are not available in a public repository because they contain protected health information and are subject to institutional and provincial privacy regulations. De‐identified aggregate data and analysis outputs may be made available from the corresponding author on reasonable request and with prior approval from BC Cancer and the University of British Columbia research ethics boards. Curated public software releases supporting the biopsy‐localisation, dosimetry, and statistical analyses are available through BiopsyContext^30^ and BiopsyContext Dosimetry.^31^
