# Supplementary material for: Gaussian process regression of HDR prostate biopsy dose for improved modelling of localization‐induced uncertainty
Source: Med Phys. 2026 Nov 1;53(11):e70712. doi: 10.1002/mp.70712 (PMC13724794; doi:10.1002/mp.70712)
Supplement: Supplementary file 1 [file MP-53-0-s001.pdf]

Supporting Information for Gaussian Process Regression of HDR Prostate Biopsy Dose for Improved Modelling of Localization-Induced Uncertainty

Matthew Muscat,[1,2] Juanita Crook,[2,3] Andrew Jirasek,[1] Jeffrey Andrews,[1] and Nathan Becker[1,2]

[1)] *Department of Computer Science, Mathematics, Physics and Statistics, Irving K. Barber Faculty of Science, University of British Columbia Okanagan, Kelowna, British Columbia, Canada, V1V 1V7*

[2)] *BC Cancer, Kelowna, British Columbia, Canada, V1Y 5L3*

[3)] *Department of Surgery, University of British Columbia, Kelowna, British Columbia, Canada, V1V 1V7*

(Dated: 26 September 2026)

This Supporting Information accompanies the main article and provides supplementary methods, extended biopsy-level results, residual diagnostics, kernel sensitivity analyses, and blocked cross-validation details.

S1. SUPPORTING METHODS AND THEORY

This section gives the complete model formulation used throughout the study: observation model and empirical semivariogram construction, Matérn hyperparameter fitting, and ordinary-mean GPR posterior inference. The resulting quantities map directly to reported outputs, including $\widehat{\ell}_b$, $\widehat{\tau}_b^2$, $\sigma_{b,v}^{\mathrm{GP}}$, standardized residuals $r_{b,v}^{\mathrm{std},m}$, and uncertainty-reduction summaries such as $\Delta_b^{(\mathrm{SD})}$. Here $m \in \{\mathrm{lat}, \mathrm{obs}\}$ indexes the predictive-variance definition in $r_{b,v}^{\mathrm{std},m}$: lat denotes the latent-only definition and obs denotes the latent-plus-localization definition. The obs superscript is used only to distinguish this two-term definition from the latent-only definition. Baseline and kernel-sensitivity residual analyses use the latent definition, while blocked cross-validation primary reporting uses the latent-plus-localization definition (equation (S31)).

S1.1. Latent dose field and observation model

For each biopsy b we model the latent dose profile along the biopsy axis as a real-valued stochastic process

$$\{D_b(z) : z \in \mathcal{I}_b\}, \tag{S1}$$

defined on the axial interval $\mathcal{I}_b$ between the entry and exit points of the core inside the prostate. Intuitively, $D_b(z)$ represents the model-conditional central-tendency dose profile underlying the voxelwise Monte Carlo summaries, rather than a perfectly localized physical truth. The Monte Carlo variability reflects rigid perturbations of the biopsy trajectory under the fixed localisation uncertainty model. The analyzed profile set comprised the nominal profile at $t = 0$ and $N_{\mathrm{MC}} = 10{,}000$ perturbed profiles, giving $T = N_{\mathrm{MC}} + 1 = 10{,}001$ profiles indexed by $t = 0, \ldots, N_{\mathrm{MC}}$. The discrete samples $\{D_{b,v}^{(t)}\}_{t=0}^{N_{\mathrm{MC}}}$ at voxel axial positions $z_{b,v}$ comprise the nominal evaluation and evaluations perturbed by rigid translations of the trajectory under the fixed localisation uncertainty model. Under this view, neighbouring voxels

are expected to be strongly correlated rather than independent along the core, consistent with the axial dependence observed in earlier finite-difference analyses.[1,2] In particular, we assume that the per-voxel Monte Carlo median

$$\widetilde{D}_{b,v} = \mathrm{median}_t\left(D_{b,v}^{(t)}\right) \tag{S2}$$

provides a robust point estimate of $D_b(z_{b,v})$, while the sample variance

$$\widehat{\sigma}^2_{b,v} = \frac{1}{T-1}\sum_{t=0}^{N_{\mathrm{MC}}}\left(D_{b,v}^{(t)} - \overline{D}_{b,v}\right)^2 \tag{S3}$$

captures local dispersion due to rigid perturbations, where $\overline{D}_{b,v}$ is the Monte Carlo mean. This voxelwise marginal variance quantifies localization-induced dose dispersion under the assumed perturbation model. It is used as a heteroscedastic observation-uncertainty term and is not the Monte Carlo sampling variance of the estimated median $\widetilde{D}_{b,v}$. Across voxels, these variances are the diagonal entries of the sample covariance matrix that would be obtained from the same trialwise dose vectors across the core; the present model retains these marginal variances but does not explicitly include the corresponding off-diagonal cross-voxel localization covariances. We then adopt the measurement model

$$\widetilde{D}_{b,v} = D_b(z_{b,v}) + \varepsilon_{b,v}, \tag{S4}$$

This is the standard latent-function plus additive-noise observation model used in GP regression.[3] As a working approximation, although the shared rigid perturbations can induce cross-voxel dependence, after conditioning on the spatially correlated latent dose profile the remaining voxelwise observation errors are modelled as independent zero-mean Gaussian terms $\varepsilon_{b,v} \sim \mathcal{N}(0, \sigma^2_{\varepsilon,b,v})$. The effective noise variance at each voxel is taken as

$$\sigma^2_{\varepsilon,b,v} = \widehat{\sigma}^2_{b,v} + \tau_b^2, \tag{S5}$$

where $\tau_b^2 \geq 0$ is a biopsy-specific nugget variance capturing micro-scale variability not resolved at the 1 mm voxel size. In semivariogram terms it appears as a non-zero intercept as $h \to 0$, and therefore absorbs micro-scale heterogeneity and residual measurement/registration mismatch not captured by the correlated component. This diagonal working observation covariance retains the voxelwise localization-induced variances but not cross-voxel localization-error covariances. It does not make the latent doses independent:

their spatial correlation is represented separately by the full Matérn covariance.[3] To quantify this axial correlation structure in a way that is directly estimable from the Monte Carlo samples, we use a biopsy-specific empirical semivariogram.

S1.2. Matérn covariance and semivariogram

In the main analysis we use an ordinary-mean formulation, writing

$$D_b(z) = m_b + g_b(z), \tag{S6}$$

where m_b is a biopsy-specific constant mean and $g_b(z)$ is a second-order stationary Gaussian process with covariance function $k_b(h)$ depending only on axial separation h:

$$g_b(z) \sim \mathcal{GP}\big(0,\, k_b(h)\big). \tag{S7}$$

Throughout we use the Matérn family in one dimension with fixed smoothness parameter ν,[4,5]

$$k_b(h) = c_b\, \kappa_\nu\!\left(\frac{|h|}{\ell_b}\right), \tag{S8}$$

where c_b is the process variance component (partial sill), $\ell_b > 0$ is an axial length-scale, and κ_ν is the Matérn correlation function

$$\kappa_\nu(r) = \frac{2^{1-\nu}}{\Gamma(\nu)} (\sqrt{2\nu}\, r)^\nu K_\nu\big(\sqrt{2\nu}\, r\big), \qquad r \geq 0, \tag{S9}$$

When an additive nugget term τ_b^2 is included (below), c_b is the partial sill (spatially correlated variance): the semivariogram rises from τ_b^2 near $h = 0$ toward $\tau_b^2 + c_b$ at large separations. Here, $\Gamma(\nu)$ is the Euler gamma function and K_ν is the modified Bessel function of the second kind. The exponential kernel is recovered at $\nu = 0.5$, while the squared-exponential (RBF/Gaussian) kernel is the smooth-limit case $\nu \to \infty$ (up to kernel parameterization). In the main analysis we set $\nu = 3/2$, selected as the baseline kernel in the main manuscript, for which

$$\kappa_{3/2}(r) = \Big(1 + \sqrt{3}\, r\Big) \exp\Big(-\sqrt{3}\, r\Big). \tag{S10}$$

For a second-order stationary process, the semivariogram-covariance identity is $\gamma_b(h) = \frac{1}{2} \operatorname{Var}[D_b(z) - D_b(z+h)] = k_b(0) - k_b(h)$,[4] so the corresponding theoretical semivariogram (excluding the nugget) is

$$\gamma_b^{(\text{Matérn})}(h) = \tfrac{1}{2}\, \mathbb{E}\big[(D_b(z) - D_b(z+h))^2\big] = c_b - k_b(h). \tag{S11}$$

To account for micro-scale variability, we introduce a nugget variance τ_b^2 which appears as an additive constant in the semivariogram. The full model semivariogram is therefore

$$\gamma_b\big(h;\, c_b, \ell_b, \tau_b^2\big) = \begin{cases} 0, & h = 0, \\ c_b - k_b(h) + \tau_b^2, & h > 0. \end{cases} \tag{S12}$$

In the discrete setting, let $h_k = k\,\text{mm}$ denote the centre of lag bin k, with bin width $\Delta h = 1\,\text{mm}$, and define the physical-distance pair set

$$\mathcal{P}_{b,k} = \left\{(i,j) : 1 \le i < j \le n_b,\ \big||z_{b,j} - z_{b,i}| - h_k\big| \le \frac{\Delta h}{2}\right\}. \tag{S13}$$

Given the Monte Carlo dose matrix for biopsy b, with entries $D_{b,v}^{(t)}$ arranged into a $T \times n_b$ array, we estimate the empirical semivariogram as

$$\widehat{\gamma}_b(h_k) = \frac{1}{2T\,|\mathcal{P}_{b,k}|} \sum_{t=0}^{N_{\text{MC}}} \sum_{(i,j)\in\mathcal{P}_{b,k}} \big(D_{b,j}^{(t)} - D_{b,i}^{(t)}\big)^2, \tag{S14}$$

which is the classical empirical semivariogram estimator in discrete form, evaluated here over physical-distance-binned voxel pairs and Monte Carlo profiles.[4] For a complete contiguous $1\,\text{mm}$ lattice, $|\mathcal{P}_{b,k}| = n_b - k$, so this pairwise form reduces to the usual contiguous-shift expression. The physical-distance form remains valid for the gapped training lattices created by blocked cross-validation. At each lag, squared dose differences are averaged over all eligible within-profile pairs without mixing dose values between profiles.

For each biopsy b, all available lag indices $k = 1, \ldots, n_b - 1$ were retained, so $k_{\max} = n_b - 1$. We estimated the Matérn hyperparameters $\theta_b = (c_b, \ell_b, \tau_b^2)$ by unweighted nonlinear least squares,

$$\widehat{\theta}_b = \arg\min_{\theta_b} \sum_{k=1}^{k_{\max}} \big[\widehat{\gamma}_b(h_k) - \gamma_b(h_k;\, \theta_b)\big]^2, \tag{S15}$$

Let $\widehat{\gamma}_{b,\max} = \max_k \widehat{\gamma}_b(h_k)$, $\widehat{\gamma}_{b,\min} = \min_k \widehat{\gamma}_b(h_k)$, $\Delta h_{b,\min}$ be the smallest retained lag increment, and $h_{b,\max}$ the largest retained lag. With semivariances expressed numerically in Gy^2 and lag distances in mm, the fitting bounds were

$$c_b \in \big[10^{-6}, \max\big(10\widehat{\gamma}_{b,\max}, 10^{-3}\big)\big], \tag{S16}$$

$$\ell_b \in [\max(\Delta h_{b,\min}, 0.05), \max(5h_{b,\max}, 1.0)], \tag{S17}$$

$$\tau_b^2 \in [0, \max(\widehat{\gamma}_{b,\max}, 1.0)]. \tag{S18}$$

These bounds are numerical fitting safeguards and do not imply that length scales extending beyond the observed lag support are physically resolved. Fits were initialized with $c_{b,0} = \max(0.7\widehat{\gamma}_{b,\max}, 10^{-3})$, $\ell_{b,0} = \max(h_{b,\max}/3, 1\,\mathrm{mm})$, and $\tau_{b,0}^2 = \max(\widehat{\gamma}_{b,\min}, 10^{-6})$, with at most 10,000 function evaluations. We also compute, for each biopsy, a root-mean-square error between the empirical and fitted semivariograms as a measure of fit quality,

$$\mathrm{RMSE}_b^{(\gamma)} = \sqrt{\frac{1}{k_{\max}} \sum_{k=1}^{k_{\max}} \left[\widehat{\gamma}_b(h_k) - \gamma_b(h_k;\, \widehat{\theta}_b)\right]^2}. \tag{S19}$$

The fitted length-scale $\widehat{\ell}_b$ characterizes the axial scale of the modelled spatial correlation of the dose field along the core, while the nugget $\widehat{\tau}_b^2$ quantifies the component of variance that the smooth Matérn model cannot attribute to spatially correlated structure.

S1.3. Gaussian-process posterior with heteroscedastic noise

For convenience, the per-biopsy inputs may be written as $\mathbf{z}_b = (z_{b,1}, \ldots, z_{b,n_b})^\top$, $\widetilde{\mathbf{D}}_b = (\widetilde{D}_{b,1}, \ldots, \widetilde{D}_{b,n_b})^\top$, and $\widehat{\boldsymbol{\sigma}}_b^2 = (\widehat{\sigma}_{b,1}^2, \ldots, \widehat{\sigma}_{b,n_b}^2)^\top$. In the GP notation below we write $\mathbf{X}_b \equiv \mathbf{z}_b$ and $\mathbf{y}_b \equiv \widetilde{\mathbf{D}}_b$. With ordinary-mean GPR, we estimate a biopsy-specific constant mean $\widehat{m}_b$ from the training data and work with centered targets $\widetilde{\mathbf{y}}_b = \mathbf{y}_b - \widehat{m}_b\mathbf{1}$. This is the ordinary-mean kriging formulation: the GP is applied to residuals about an estimated constant mean, and $\widehat{m}_b$ is added back to the posterior mean prediction. Given fitted hyperparameters $\widehat{\theta}_b = (\widehat{c}_b, \widehat{\ell}_b, \widehat{\tau}_b^2)$, the $n_b \times n_b$ covariance matrix between training points has entries determined by the fitted semivariogram (equivalently, the fitted Matérn covariance; baseline $\nu = 3/2$)[3,4], where $\widehat{c}_b$ is the fitted partial sill (the spatially correlated variance component), whereas $\widehat{\sigma}_{b,v}^2$ is the voxelwise Monte Carlo input variance appearing in the noise model,

$$\left[\mathbf{K}_{bb}\right]_{ij} = k_b\big(z_{b,i} - z_{b,j};\, \widehat{\theta}_b\big), \tag{S20}$$

and the effective noise covariance is taken as

$$\boldsymbol{\Sigma}_{\varepsilon,b} = \mathrm{diag}\big(\widehat{\sigma}_{b,1}^2, \ldots, \widehat{\sigma}_{b,n_b}^2\big) + \widehat{\tau}_b^2\, \mathbf{I}_{n_b}. \tag{S21}$$

This diagonal term is a working conditional-independence approximation for the localization-induced observation component: it retains each voxel's marginal localization-induced variance but not covariance between voxelwise observation errors. The latent covariance $\mathbf{K}_{bb}$

remains a full spatial covariance across all voxel locations. Intuitively, this heteroscedastic form allows each voxel to retain its own Monte Carlo uncertainty level along the core, rather than forcing a single shared noise variance for all axial positions. Voxels with larger $\widehat{\sigma}^2_{b,v}$ are therefore treated as less reliable observations in the posterior update. Defining $\mathbf{C}_b = \mathbf{K}_{bb} + \mathbf{\Sigma}_{\varepsilon,b}$, the ordinary-kriging constant mean is estimated by generalized least squares (GLS),

$$\widehat{m}_b = \frac{\mathbf{1}^\top \mathbf{C}_b^{-1} \mathbf{y}_b}{\mathbf{1}^\top \mathbf{C}_b^{-1} \mathbf{1}}, \tag{S22}$$

Here $k_b(h)$ is the scalar covariance function, $\mathbf{K}_{bb}$ is its discretization on training locations, and $\mathbf{C}_b$ is the noisy training covariance used for posterior conditioning. After this, inference is performed on residual targets $\widetilde{\mathbf{y}}_b = \mathbf{y}_b - \widehat{m}_b \mathbf{1}$, and $\widehat{m}_b$ is added back to posterior mean predictions[3,4]. This follows directly from the latent-function plus additive-noise observation model introduced above: under the working conditional-independence approximation for $\varepsilon_{b,v}$, the observation-error covariance is diagonal with per-voxel variance $\widehat{\sigma}^2_{b,v} + \widehat{\tau}^2_b$[3]. For numerical stability, voxelwise observation variances entering the GP were floored at $10^{-6}\,\mathrm{Gy}^2$, and a fixed diagonal jitter of $10^{-8}\,\mathrm{Gy}^2$ was added before Cholesky factorization. The jitter was a numerical safeguard and was not treated as a model uncertainty term.

For any set of q_b prediction locations $\mathbf{X}_b^\star = (z_1^\star, \ldots, z_{q_b}^\star)^\top$ along the core, the joint prior distribution of the centered latent field $g_b(z)$ at training and prediction points is

$$\begin{pmatrix} \mathbf{g}_b \\ \mathbf{g}_b^\star \end{pmatrix} \sim \mathcal{N}\left(\begin{pmatrix} \mathbf{0} \\ \mathbf{0} \end{pmatrix}, \begin{pmatrix} \mathbf{K}_{bb} & \mathbf{K}_{b\star} \\ \mathbf{K}_{\star b} & \mathbf{K}_{\star\star} \end{pmatrix} \right), \tag{S23}$$

the standard joint Gaussian prior construction for GP regression[3], where $\mathbf{g}_b$ and $\mathbf{g}_b^\star$ denote centered latent-field values at $\mathbf{X}_b$ and $\mathbf{X}_b^\star$, respectively, $\mathbf{K}_{\star b}$ is the $q_b \times n_b$ covariance between prediction and training locations, $\mathbf{K}_{b\star} = \mathbf{K}_{\star b}^\mathsf{T}$, and $\mathbf{K}_{\star\star}$ is the $q_b \times q_b$ covariance among prediction locations. Elementwise, these entries are kernel evaluations at pairwise axial separations (e.g., $[\mathbf{K}_{bb}]_{ij} = k_b(z_{b,i} - z_{b,j}; \widehat{\theta}_b)$ and $[\mathbf{K}_{\star b}]_{ui} = k_b(z_u^\star - z_{b,i}; \widehat{\theta}_b)$). Consequently, each posterior prediction is a covariance-weighted combination of all training observations: the fitted semivariogram parameters determine the covariance weights through k_b, while $(\mathbf{K}_{bb} + \mathbf{\Sigma}_{\varepsilon,b})^{-1}$ accounts for training-point covariance and heteroscedastic noise.

Under this working conditionally independent Gaussian observation-error model, conditioning the latent field with a known mean gives the usual Gaussian-process covariance

term from covariance blocks $\mathbf{K}_{bb} + \mathbf{\Sigma}_{\varepsilon,b}$, $\mathbf{K}_{\star b}$, and $\mathbf{K}_{\star\star}$. Because the biopsy-specific constant mean is unknown and estimated by GLS, the ordinary-kriging prediction-error covariance additionally contains the standard mean-estimation correction.[3,4] With $\mathbf{1}$ and $\mathbf{1}_\star$ denoting vectors of ones at the training and prediction locations, respectively, define

$$\mathbf{u}_\star = \mathbf{1}_\star - \mathbf{K}_{\star b}\mathbf{C}_b^{-1}\mathbf{1}. \tag{S24}$$

$$\boldsymbol{\mu}_b^{\mathrm{GP}}(\mathbf{X}_b^\star) = \widehat{m}_b \mathbf{1}_\star + \mathbf{K}_{\star b}\mathbf{C}_b^{-1}\widetilde{\mathbf{y}}_b, \tag{S25}$$

$$\mathbf{\Sigma}_b^{\mathrm{GP}}(\mathbf{X}_b^\star) = \mathbf{K}_{\star\star} - \mathbf{K}_{\star b}\mathbf{C}_b^{-1}\mathbf{K}_{b\star} + \frac{\mathbf{u}_\star \mathbf{u}_\star^\mathsf{T}}{\mathbf{1}^\mathsf{T}\mathbf{C}_b^{-1}\mathbf{1}}, \tag{S26}$$

For one prediction location $z^\star$, with $\mathbf{C}_b$ defined above and $\widetilde{y}_{b,j} = y_{b,j} - \widehat{m}_b$, let $\mathbf{k}_{\star b} = \big(k_b(z^\star - z_{b,1}; \widehat{\theta}_b), \ldots, k_b(z^\star - z_{b,n_b}; \widehat{\theta}_b)\big)^\top$. The elementwise posterior mean and latent variance are

$$\mu_b^{\mathrm{GP}}(z^\star) = \widehat{m}_b + \sum_{i=1}^{n_b}\sum_{j=1}^{n_b} k_b(z^\star - z_{b,i}; \widehat{\theta}_b)\,[\mathbf{C}_b^{-1}]_{ij}\,\widetilde{y}_{b,j}, \tag{S27}$$

$$\big(\sigma_b^{\mathrm{GP}}(z^\star)\big)^2 = k_b(0;\widehat{\theta}_b) - \sum_{i=1}^{n_b}\sum_{j=1}^{n_b} k_b(z^\star - z_{b,i}; \widehat{\theta}_b)\,[\mathbf{C}_b^{-1}]_{ij}\,k_b(z_{b,j} - z^\star; \widehat{\theta}_b) + \frac{\big[1 - \mathbf{k}_{\star b}^\mathsf{T}\mathbf{C}_b^{-1}\mathbf{1}\big]^2}{\mathbf{1}^\mathsf{T}\mathbf{C}_b^{-1}\mathbf{1}}, \tag{S28}$$

The final term accounts for uncertainty from estimating the biopsy-specific constant mean.[3,4] This ordinary-mean correction was included in the posterior calculations used for all reported results. Posterior variances are conditional on the fitted semivariogram hyperparameters and do not propagate uncertainty in their estimation. These expressions can be evaluated directly once $\widehat{\theta}_b$, $\widehat{m}_b$, and $\mathbf{\Sigma}_{\varepsilon,b}$ are known. Evaluating these expressions at $\mathbf{X}_b^\star = \mathbf{X}_b$ yields voxelwise posterior mean and latent variance. In practice, these quantities are also evaluated on a finer axial grid to visualize smooth dose profiles with pointwise credible bands,

$$\mu_{b,v}^{\mathrm{GP}} = \mu_b^{\mathrm{GP}}(z^\star)\big|_{z^\star = z_{b,v}} = \mathbb{E}\big[D_b(z_{b,v}) \,\big|\, \mathbf{y}_b\big], \tag{S29}$$

$$\big(\sigma_{b,v}^{\mathrm{GP}}\big)^2 = \big(\sigma_b^{\mathrm{GP}}(z^\star)\big)^2\big|_{z^\star = z_{b,v}} = \mathrm{Var}\big[D_b(z_{b,v}) \,\big|\, \mathbf{y}_b\big]. \tag{S30}$$

For primary blocked-CV residual standardization, the latent-plus-localization variance was

$$\big(\sigma_{b,v}^{\mathrm{pred}}\big)^2 = \big(\sigma_{b,v}^{\mathrm{pred,obs}}\big)^2 = \big(\sigma_{b,v}^{\mathrm{GP}}\big)^2 + \widehat{\sigma}_{b,v}^2, \tag{S31}$$

The paired latent-only definition was $\left(\sigma_{b,v}^{\mathrm{pred,lat}}\right)^2 = \left(\sigma_{b,v}^{\mathrm{GP}}\right)^2$. The former adds the voxelwise localization-induced dose variance; this is not the sampling variance of the Monte Carlo median. The fitted nugget variance enters GP training and conditioning but is not added as a separate term in this blocked-CV denominator. Here $\sigma_{b,v}^{\mathrm{GP}} \equiv \sigma_{b,v}^{\mathrm{pred,lat}}$ denotes the latent GP posterior standard deviation, whereas $\widehat{\sigma}_{b,v}$ denotes the voxelwise localization-induced dose standard deviation. In the analyses reported in this paper, baseline and kernel-sensitivity residual diagnostics use the latent definition, while blocked-CV primary reporting uses the latent-plus-localization definition. The latent-only definition was retained in blocked cross-validation as a paired sensitivity analysis. These voxelwise quantities are used in the main text to quantify uncertainty reduction and construct residual diagnostics.

S2. SUPPORTING PRIMARY RESULTS

S2.1. Additional semivariogram fits

To complement the representative biopsies in the main Results section, figure S1 shows additional biopsy-level semivariograms and fitted Matérn curves. The panels show clear between-biopsy heterogeneity in axial covariance structure, including both shorter- and longer-range rise patterns and varying nugget behavior near small lags. Despite this heterogeneity, the fitted curves capture the dominant empirical trend across lag in each panel, with deviations concentrated mainly at sparse large-lag points. This supports the use of biopsy-specific fitted hyperparameters within a common kernel family.

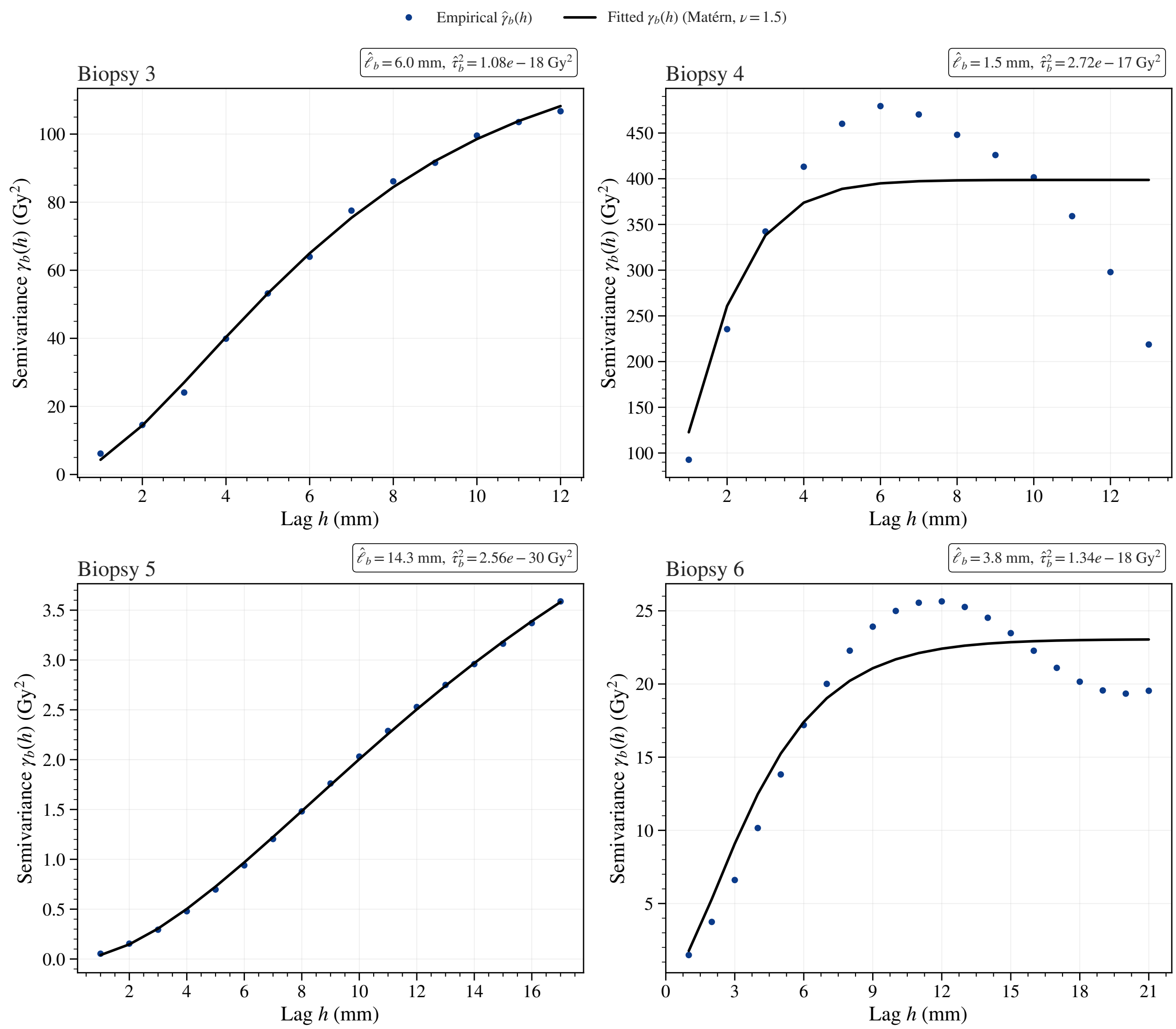

FIG. S1 Biopsy-level semivariograms and Matérn fits for a subset of biopsies. Each panel shows an empirical semivariogram with the corresponding fitted Matérn model.

S2.2. Additional dose profiles

The corresponding profile reconstructions are shown in figure S2. Across this additional subset, the GP posterior mean follows the axial dose trend while preserving biopsy-specific shape differences, and posterior uncertainty bands remain smooth compared with voxelwise Monte Carlo variability. The range of profile shapes and uncertainty envelopes is consistent with the main-result examples, indicating those examples are not isolated cases.

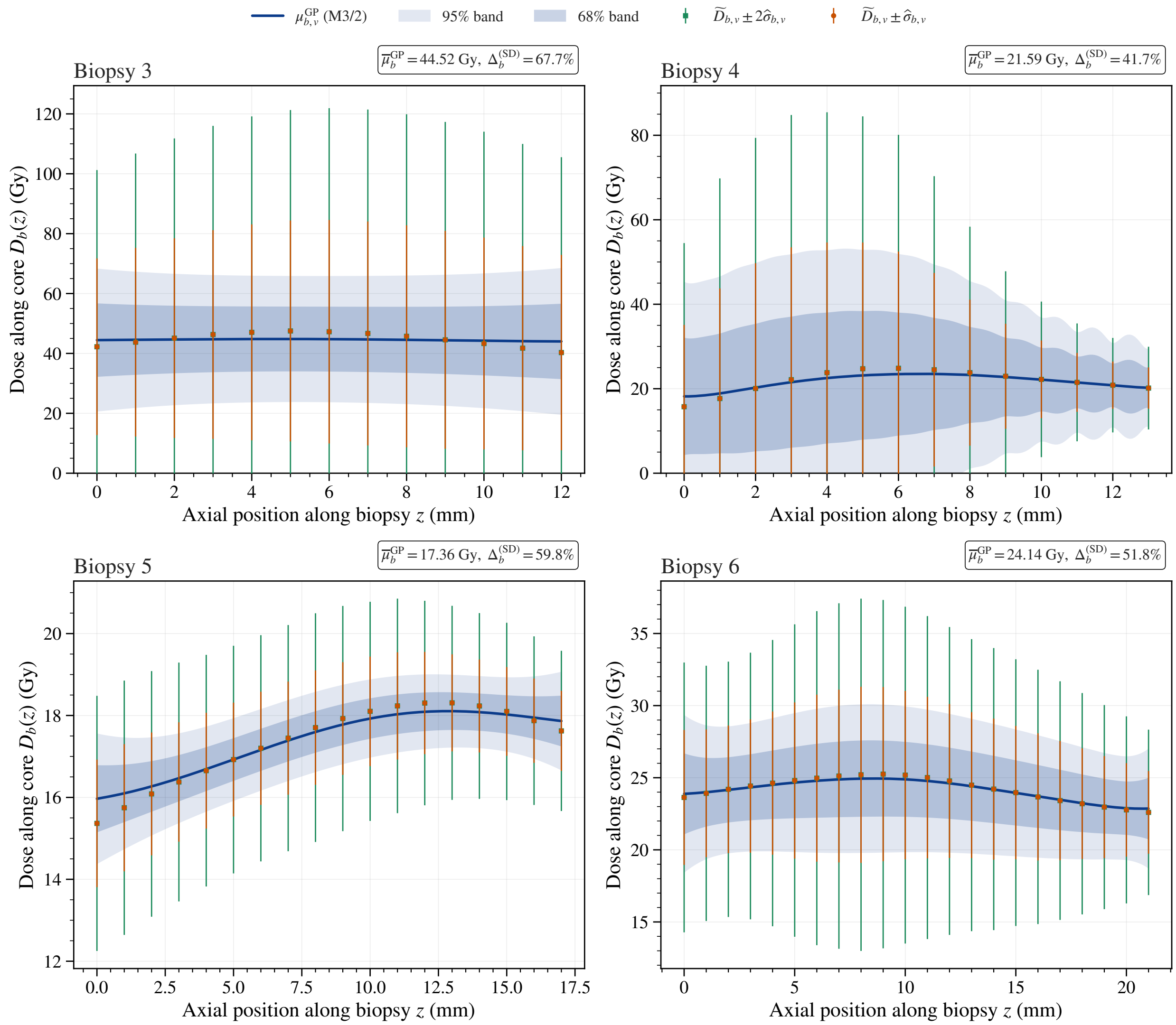

FIG. S2 Gaussian process (GP) biopsy-level profiles for a subset of biopsies. Each panel shows GP profiles along the biopsy core corresponding to the semivariogram analyses in figure S1.

S2.3. Distributions of semivariogram RMSEs and mean reduction ratios

Figure S3 summarizes cohort-level distributions for semivariogram fit error and biopsy-level uncertainty-reduction ratio. The semivariogram RMSE distribution is right-skewed with median $\mathrm{RMSE}_b^{(\gamma)} = 1.45\,\mathrm{Gy}^2$, indicating good typical fit quality with a small number of higher-error biopsies. The biopsy-level reduction-ratio distribution has median $\overline{R}_b = 2.45$, and all displayed values are > 1, confirming that GP uncertainty is lower than voxelwise Monte Carlo uncertainty at cohort scale. Together, these distributions support the main-body interpretation that the selected baseline kernel provides consistent uncertainty con-

traction while accommodating biopsy-level heterogeneity in semivariogram fit quality.

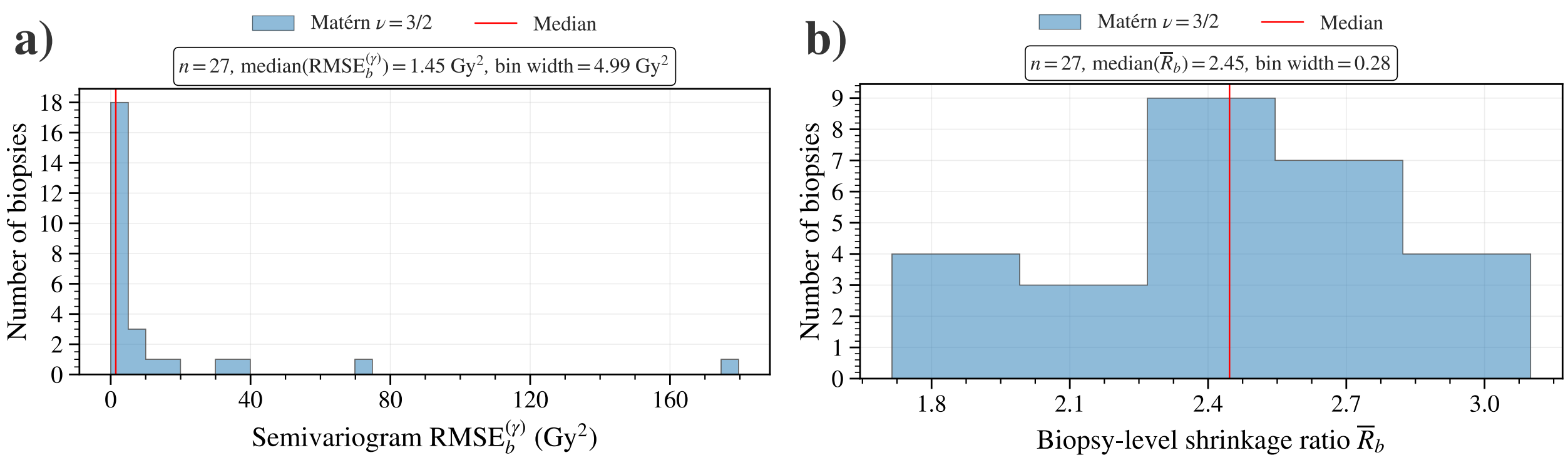

FIG. S3 Cohort distributions of (a) semivariogram fit RMSE and (b) biopsy-level shrinkage ratio $\overline{R}_b$.

S3. SUPPORTING RESIDUAL DIAGNOSTICS

This section documents descriptive latent-profile residual diagnostics for the baseline Matérn ($\nu = 1.5$) analysis. It reports biopsy-level residual behavior for the two representative main-result cases and then cohort-level summaries used to describe residual centering and spread.

S3.1. Main result biopsies (Biopsies 1 and 2)

For Biopsy 1 and Biopsy 2, figures S4 and S5 show residual traces and residual histograms under the latent standardization definition. Both biopsies show near-centered residuals with subunit spread (Biopsy 1: mean($r_{b,v}^{\text{std}}$) = 0.05, SD($r_{b,v}^{\text{std}}$) = 0.12; Biopsy 2: mean($r_{b,v}^{\text{std}}$) = 0.02, SD($r_{b,v}^{\text{std}}$) = 0.15), and both show 100% of residuals within $|r_{b,v}^{\text{std}}| \leq 1$ in the displayed summaries. These are descriptive in-sample diagnostics of the reconstructed latent profile rather than predictive-interval calibration tests.

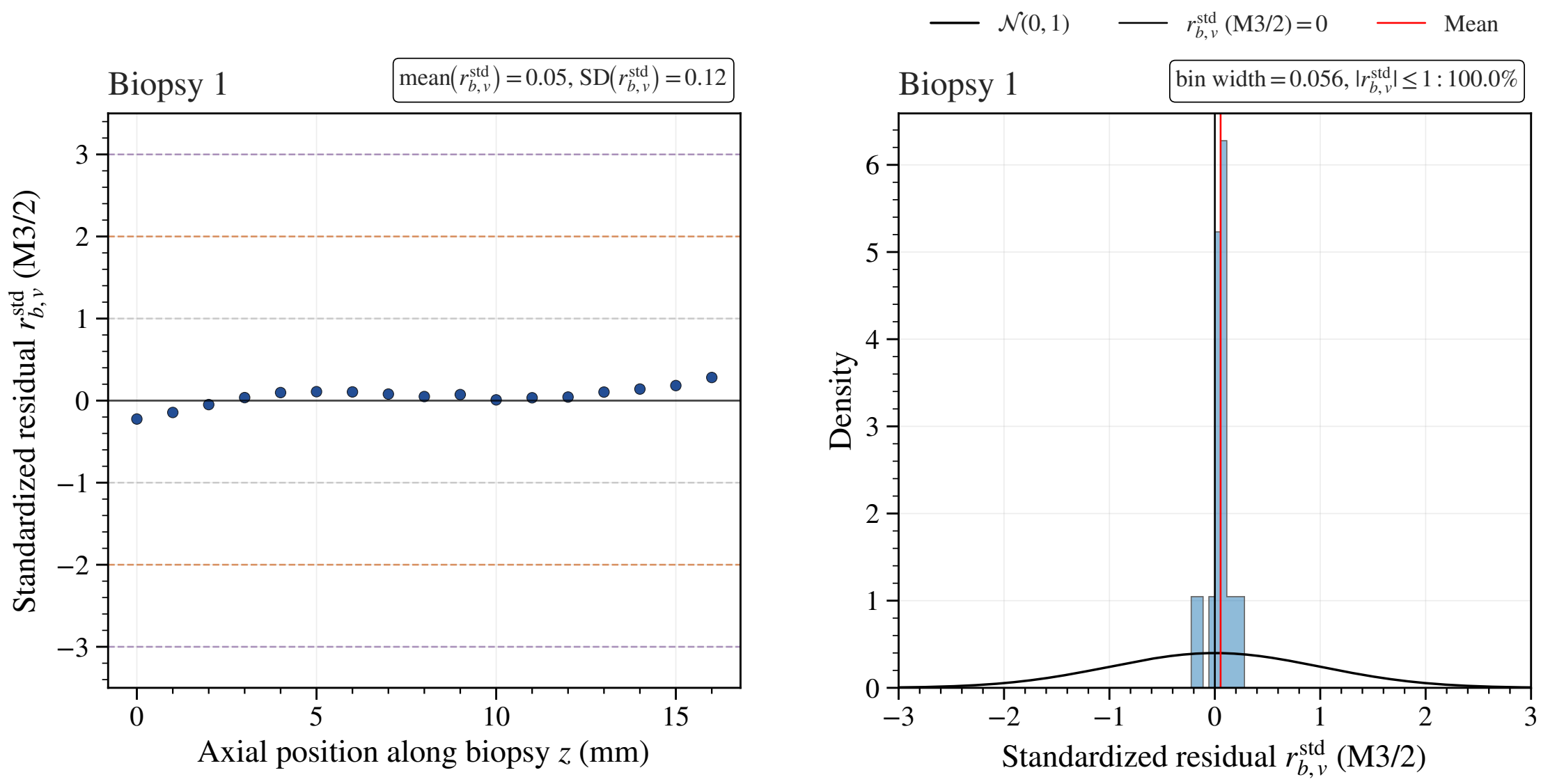

Fig. S4 Latent-profile residual diagnostics for representative biopsy 1. The left panel shows standardized residuals $r^{\mathrm{std}}_{b,v}$ vs axial coordinate, with reference lines at 0, ±2, and ±3. The right panel shows a histogram of $r^{\mathrm{std}}_{b,v}$. The residuals are approximately centered and markedly narrower than a standard normal distribution, consistent with conservative uncertainty scaling for this representative biopsy; this diagnostic does not by itself establish Gaussianity.

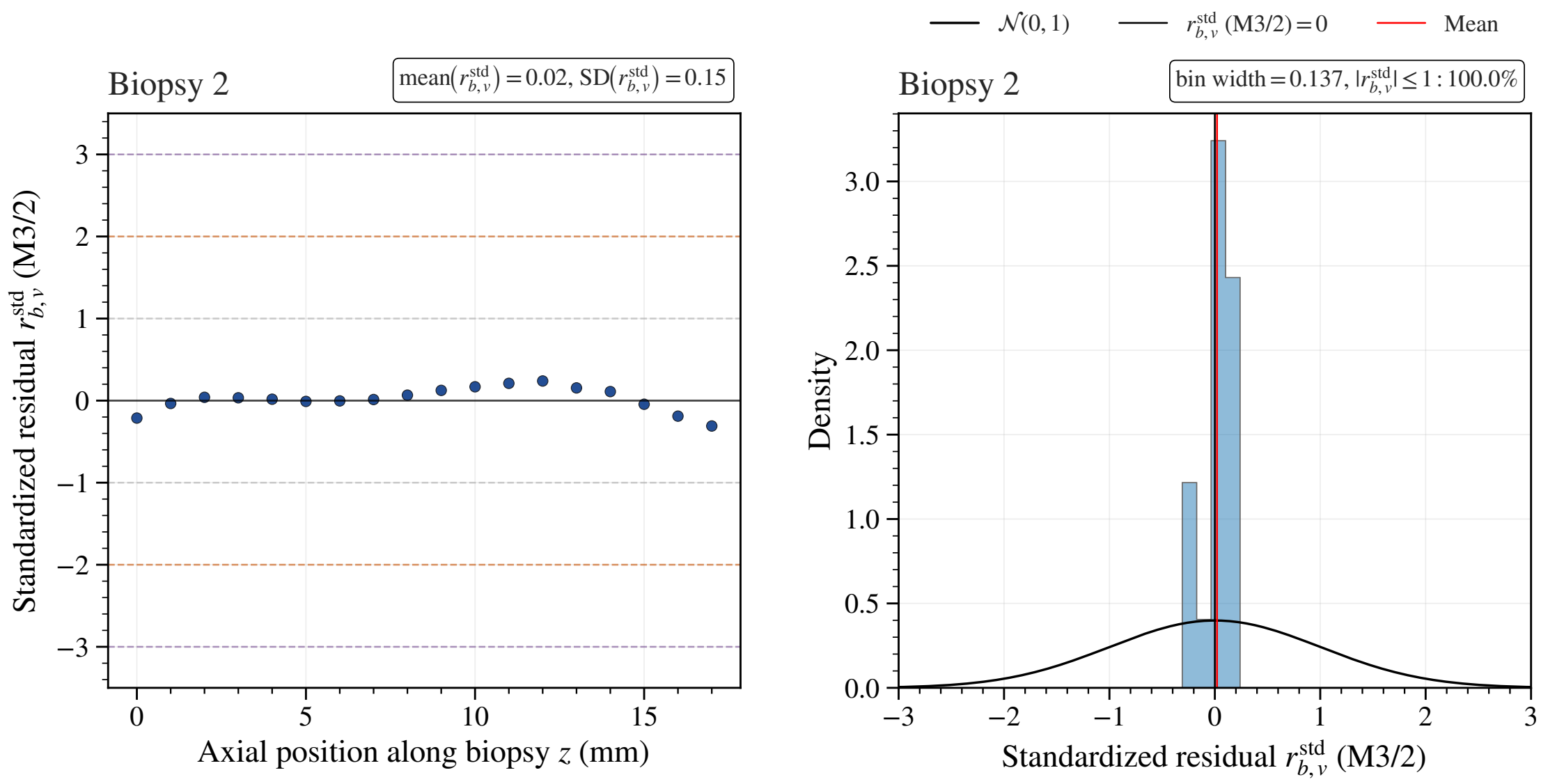

FIG. S5 Latent-profile residual diagnostics for representative biopsy 2, illustrating the variability in residual patterns across the cohort. The left panel shows standardized residuals $r^{\mathrm{std}}_{b,v}$ vs axial coordinate, while the right panel shows a histogram of $r^{\mathrm{std}}_{b,v}$, highlighting the narrow residual spread relative to the standard-normal reference.

S3.2. Cohort

At cohort level, table S1 and figure S6 show the same pattern: residual means are close to zero (median mean($r^{\mathrm{std}}_{b,v}$) = 0.052) while residual spread remains well below the standard-normal reference value of one (median SD($r^{\mathrm{std}}_{b,v}$) = 0.157). Coverage metrics show 100% within ±1 and ±2, and 0% beyond ±3 at biopsy-median level. These residual diagnostics describe a near-centered in-sample latent residual distribution with subunit spread. Because the same voxel summaries contribute to model fitting and these diagnostics, the values are not interpreted as formal predictive calibration or as a test of the Gaussian observation-error assumption.

**Biopsy-level metric**	**Median** (Q_{25}–Q_{75})
$\mathrm{mean}\left(r^{\mathrm{std}}_{b,v}\right)$	0.052 (0.025–0.079)
$\mathrm{SD}\left(r^{\mathrm{std}}_{b,v}\right)$	0.157 (0.107–0.310)
$\%\left\{\lvert r^{\mathrm{std}}_{b,v}\rvert \le 1\right\}$	100.0% (100.0%–100.0%)
$\%\left\{\lvert r^{\mathrm{std}}_{b,v}\rvert \le 2\right\}$	100.0% (100.0%–100.0%)
$\%\left\{\lvert r^{\mathrm{std}}_{b,v}\rvert \ge 3\right\}$	0.0% (0.0%–0.0%)

Table S1 Cohort summary of biopsy-level residual diagnostics for the baseline Matérn covariance model ($\nu = 1.5$). Entries report the median and interquartile range (Q_{25}–Q_{75}) over $B = 27$ biopsies for summaries of the standardized residuals $r^{\mathrm{std}}_{b,v}$ defined in the main manuscript.

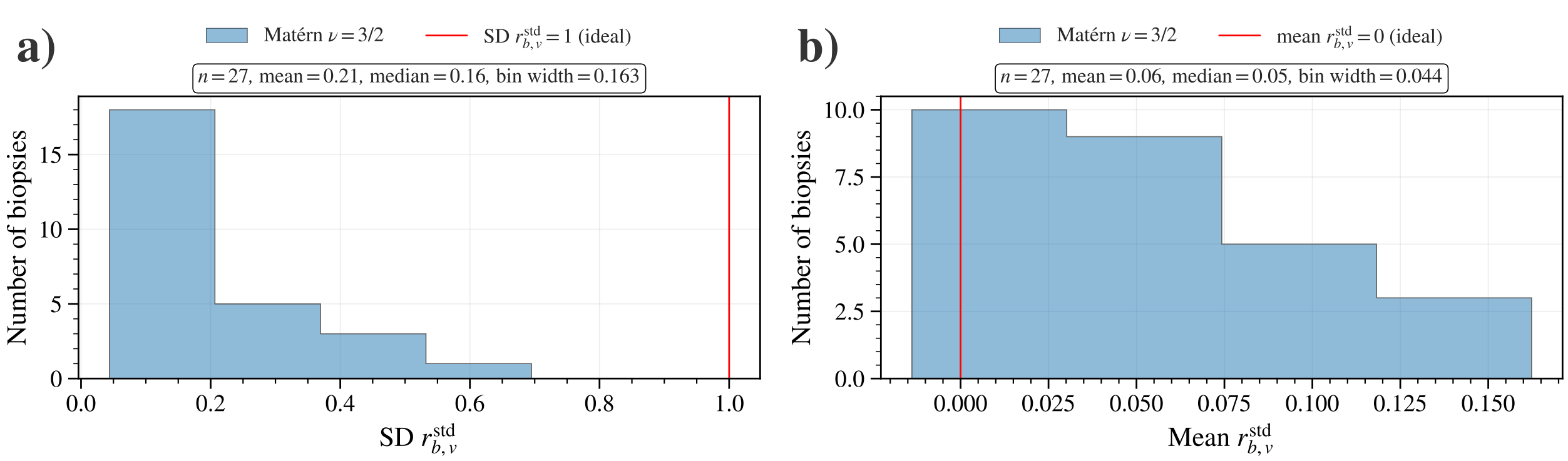

Fig. S6 Cohort-level latent-profile residual diagnostics across $B = 27$ biopsies. Panel (a) shows the distribution of SD($r^{\mathrm{std}}_{b,v}$) with the standard-normal reference value of one; panel (b) shows the distribution of mean($r^{\mathrm{std}}_{b,v}$) with the reference value of zero.

S4. SUPPORTING KERNEL SENSITIVITY ANALYSES

This section summarizes why kernel selection was not based on a single diagnostic. Latent residual behavior is compared across kernels, followed by semivariogram-fit and uncertainty-comparison summaries, and then interpreted jointly with blocked-CV predictive metrics reported in the main text.

S4.1. Residuals

Latent residual sensitivity is shown in figures S7 and S8. All kernels exhibit near-zero to mildly positive mean($r_{b,v}^{\mathrm{std}}$) with subunit SD($r_{b,v}^{\mathrm{std}}$). The near-reference-count summary in figure S7 is low for all kernels (1/27 for Matérn $\nu = 1.5$, 2/27 for Matérn $\nu = 2.5$, 3/27 for RBF, 0/27 for exponential), so latent residual behaviour alone does not provide a decisive ranking.

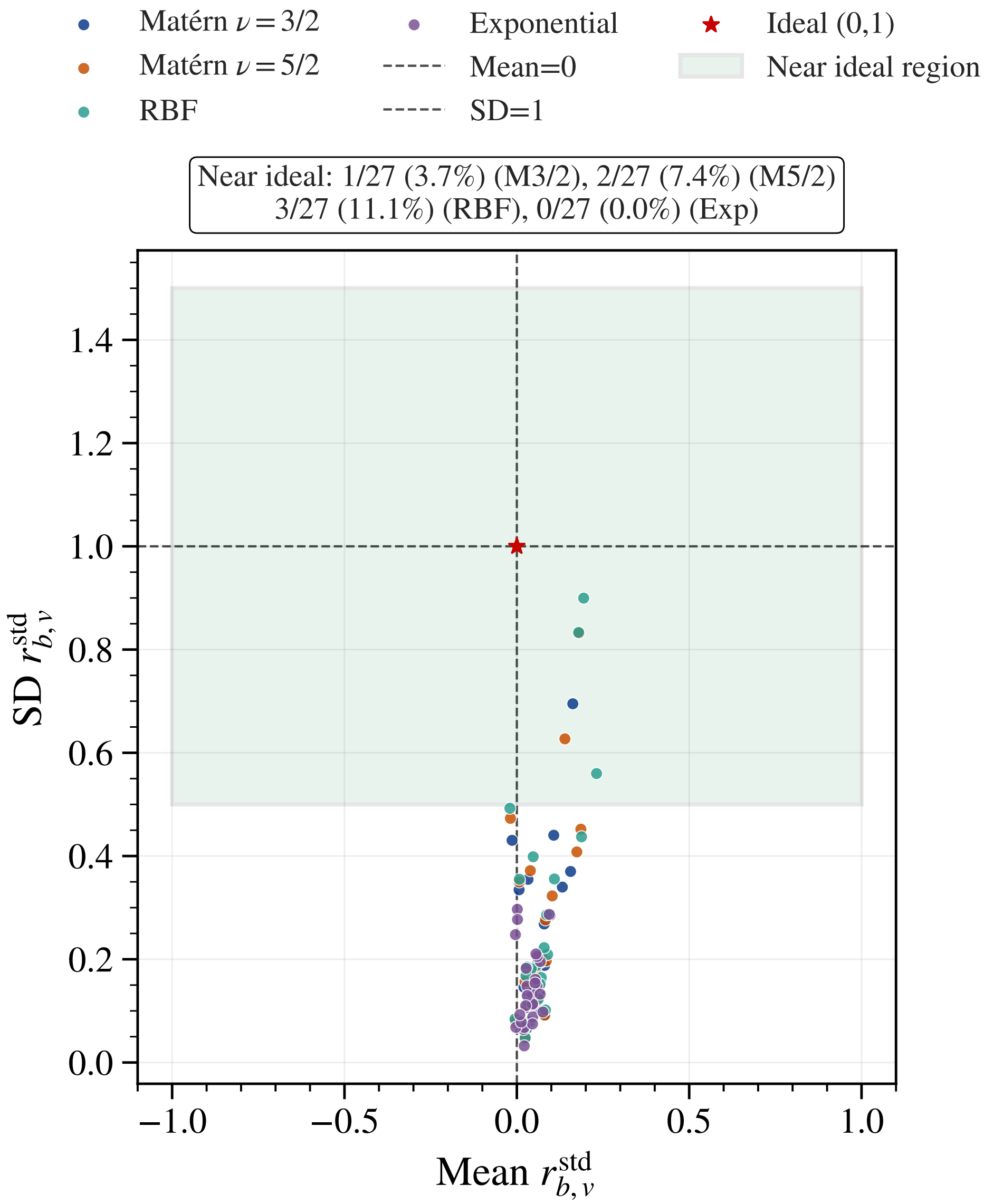

Fig. S7 Kernel sensitivity of latent-profile residuals from biopsy-level mean-SD residual scatter. Across kernels, residual means are mildly positive and residual standard deviations remain below the $(0, 1)$ reference point. This diagnostic was used jointly with blocked-CV predictive metrics to select the baseline Matérn ($\nu = 1.5$) model.

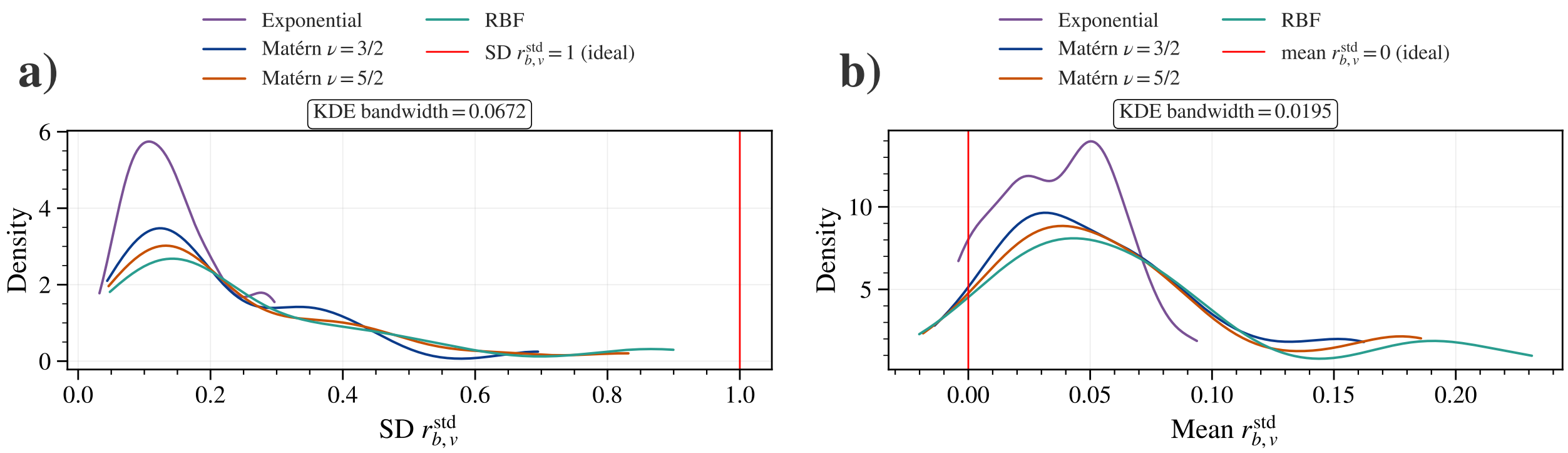

Fig. S8 Kernel sensitivity of residual moments. Panel (a) shows the biopsy-level standard deviation of standardized residuals for each kernel, with the standard-normal reference value of one; panel (b) shows the corresponding mean, with the reference value of zero. Across kernels, residuals show mildly positive means and subunit standard deviations. This figure is interpreted jointly with semivariogram-fit and blocked-CV metrics for final kernel selection.

S4.2. Semivariogram RMSE, fitted kernel length scale and MC vs. GP uncertainty comparison metrics

Output-sensitivity behavior is shown in figures S9 and S10. The semivariogram-RMSE distributions overlap, but the exponential kernel shows a heavier high-error tail than the Matérn/RBF kernels (figure S9, panel (a)). Fitted kernel length-scale distributions are kernel dependent, with exponential shifted toward longer fitted $\widehat{\ell}_b$ values (figure S9, panel (b)). Mean uncertainty-reduction-ratio distributions are also kernel dependent: exponential is shifted lower, while Matérn and RBF are shifted higher (figure S10, panel (a)). In the MC-vs-GP SD scatter (figure S10, panel (b)), all fitted lines remain below identity (uncertainty contraction across kernels), but the separation between kernels is modest relative to cohort spread. Taken together, these outputs support using blocked-CV predictive metrics to finalize baseline selection, yielding Matérn ($\nu = 1.5$).

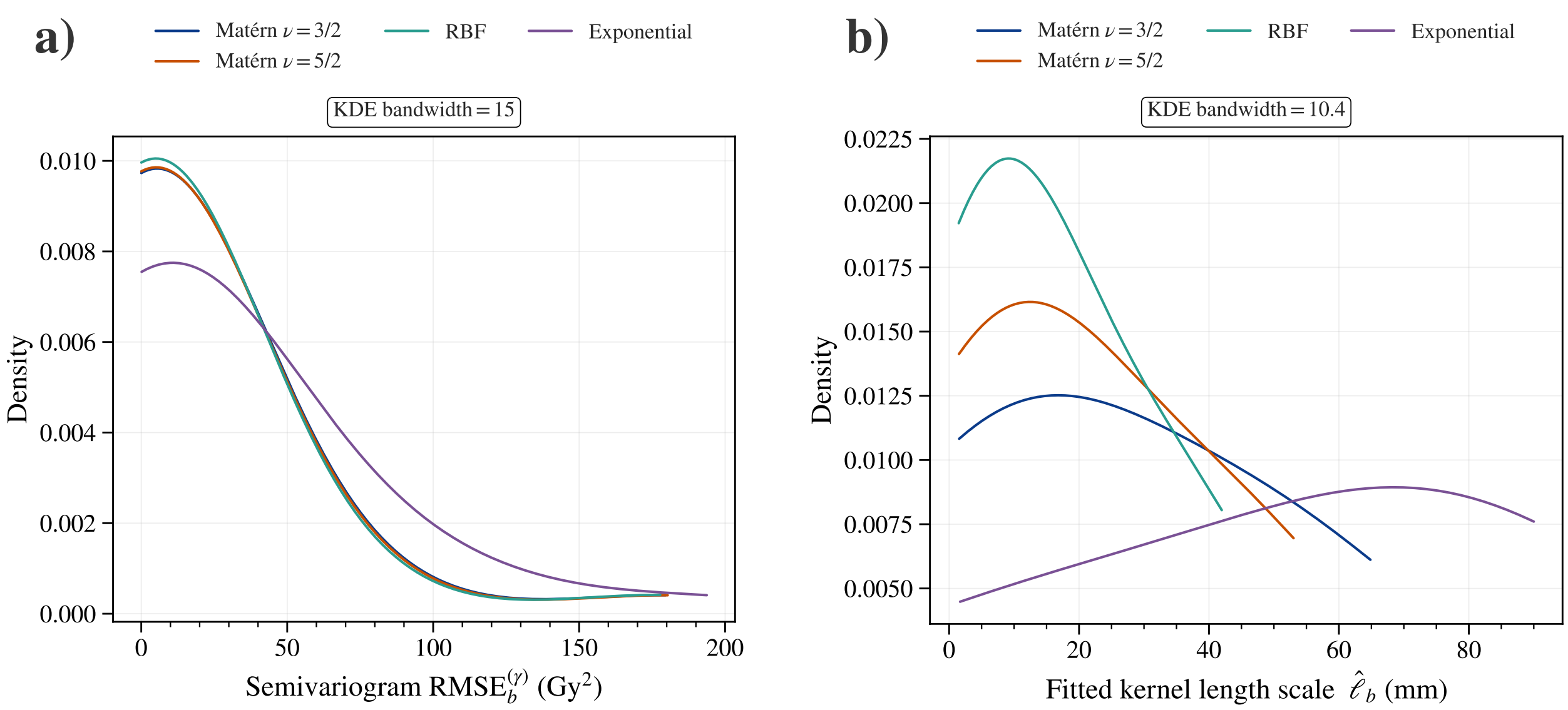

FIG. S9 Sensitivity to covariance-kernel choice of (a) semivariogram RMSE and (b) fitted kernel length scale. Each panel stratifies biopsies by kernel family.

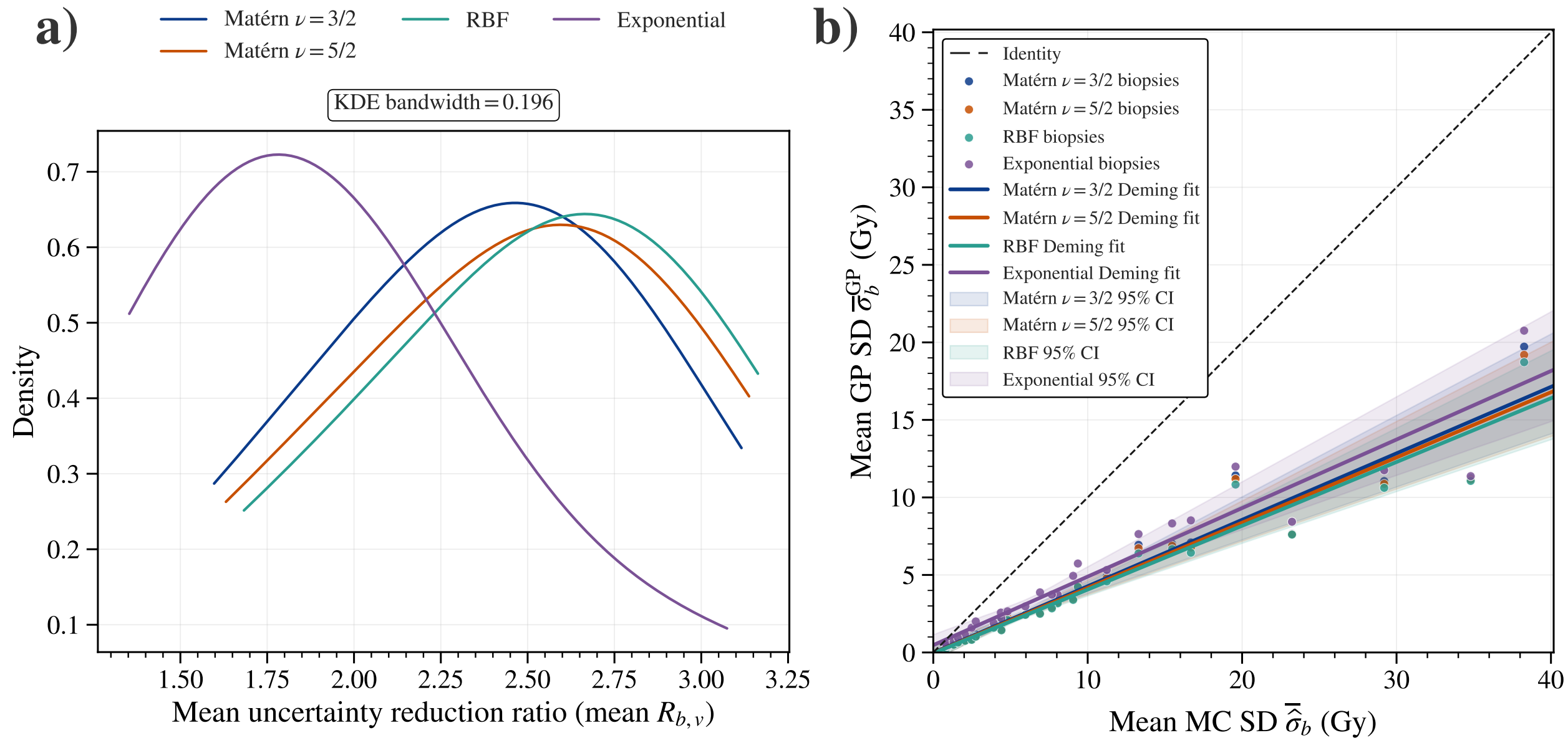

FIG. S10 Sensitivity to covariance-kernel choice of (a) mean shrinkage ratio and (b) mean Monte Carlo versus mean GPR standard deviation with associated Deming regressions. Each panel stratifies biopsies by kernel family.

S5. SUPPORTING BLOCKED CROSS-VALIDATION RESULTS

This section provides held-out validation evidence under contiguous axial blocking. It documents the fold protocol, kernel-level held-out performance summaries, sensitivity to predictive-variance definition, and representative fold-level examples used to interpret the cohort tables.

S5.1. Protocol

Blocked cross-validation was performed along each biopsy axis using contiguous held-out segments with target length 8.0 mm. Partitions were adjusted when necessary to avoid very short held-out segments, requiring each held-out segment to contain at least 3 voxels and span at least 5.0 mm. At least two evaluable folds were retained per biopsy, with partitions rebalanced to two folds when required. Consequently, some realized held-out segments exceeded 8.0 mm. For each fold, model fitting used only training voxels and predictions were evaluated only on held-out voxels. The blocked-CV analysis used ordinary-mean GPR with the median Monte Carlo target, the latent-plus-localization primary predictive-variance

definition, and a paired latent-only sensitivity definition. Kernel families evaluated were exponential, Matérn ($\nu = 1.5$), Matérn ($\nu = 2.5$), and RBF over $B = 27$ biopsies.

To keep fold-specific quantities distinct from full-biopsy fits, blocked-CV figure annotations use a fold index f. For each fold, semivariogram hyperparameters $(\widehat{c}_{b,-f}, \widehat{\ell}_{b,-f}, \widehat{\tau}^2_{b,-f})$ are refit using train-only voxels; figure annotations report

$$\widehat{\ell}_{b,-f}, \qquad \widehat{\tau}^2_{b,-f}, \tag{S32}$$

where $-f$ denotes fitting on all voxels in biopsy b except the held-out fold f. For held-out profile uncertainty reduction, we report

$$\Delta^{(\mathrm{SD,test,lat})}_{b,f} = 100 \left(1 - \frac{\overline{\sigma}^{\mathrm{GP,lat,test}}_{b,f}}{\overline{\widehat{\sigma}}^{\mathrm{test}}_{b,f}} \right), \tag{S33}$$

where $\overline{\widehat{\sigma}}^{\mathrm{test}}_{b,f} = \frac{1}{n^{\mathrm{test}}_{b,f}} \sum_{v \in \mathcal{V}^{\mathrm{test}}_{b,f}} \widehat{\sigma}_{b,v}$, $\overline{\sigma}^{\mathrm{GP,lat,test}}_{b,f} = \frac{1}{n^{\mathrm{test}}_{b,f}} \sum_{v \in \mathcal{V}^{\mathrm{test}}_{b,f}} \sigma^{\mathrm{GP}}_{b,v}$, and $n^{\mathrm{test}}_{b,f}$ denotes the number of held-out voxels contributing to fold f. This notation is used in blocked-CV profile and semivariogram panels throughout the supporting document.

S5.2. Cohort summary (held-out points only)

Held-out cohort summaries for the four candidate kernels are reported below. Among the four kernels, Matérn ($\nu = 1.5$) gives the lowest held-out point-prediction errors (median RMSE = 1.99 Gy and median MAE = 1.58 Gy), while NLPD is similar across kernels ($\approx$ 3.26 to 3.28). Residual summaries under the latent-plus-localization definition are broadly similar across kernels ($\mathrm{SD}(r^{\mathrm{std,obs}}_{b,v}) \approx 0.20$ to 0.22, with 100% within ± 1 and ± 2 at biopsy-median level), so kernel discrimination in blocked-CV is primarily driven by RMSE/MAE, not residual spread.

S5.3. Variance-definition sensitivity (latent-plus-localization vs latent-only)

For blocked-CV residual diagnostics, the standardized residuals $r^{\mathrm{std},m}_{b,v} = r_{b,v} / \sigma^{\mathrm{pred},m}_{b,v}$, with $m \in \{\mathrm{lat}, \mathrm{obs}\}$, were evaluated under the two variance definitions in equation (S31). Deltas reported below are defined as latent-plus-localization minus latent-only values for each biopsy and kernel. As shown in table S2, all kernels have negative $\Delta\,\mathrm{SD}(r^{\mathrm{std}}_{b,v})$, near-zero $\Delta\,\mathrm{mean}(r^{\mathrm{std}}_{b,v})$, and positive ΔNLPD. Negative $\Delta\,\mathrm{SD}(r^{\mathrm{std}}_{b,v})$ values indicate that including $\widehat{\sigma}^2_{b,v}$

Table S2 Biopsy-level variance-definition deltas under blocked cross-validation, reported as median (Q_{25}, Q_{75}) over paired biopsies. Deltas are defined as latent-plus-localization minus latent-only variance definition: $\Delta x = x_{\text{obs}} - x_{\text{lat}}$.

**Kernel**	$\Delta\,\text{mean}(r_{b,v}^{\text{std}})$	$\Delta\,\text{SD}(r_{b,v}^{\text{std}})$	ΔNLPD
Exp	-0.019 (-0.150, -0.005)	-0.128 (-0.279, -0.056)	0.256 (0.085, 0.401)
Matérn $\nu = 1.5$	-0.012 (-0.100, 0.002)	-0.087 (-0.242, -0.045)	0.293 (0.149, 0.399)
Matérn $\nu = 2.5$	-0.012 (-0.097, 0.002)	-0.092 (-0.241, -0.046)	0.306 (0.163, 0.404)
RBF	-0.014 (-0.098, -0.000)	-0.099 (-0.249, -0.047)	0.313 (0.171, 0.406)

in the latent-plus-localization definition yields wider $\sigma_{b,v}^{\text{pred}}$ and therefore more conservative standardized-residual spread, while the positive ΔNLPD indicates that, for these held-out residuals, the increase in the log-variance contribution outweighed the reduction in the squared-residual contribution.

S5.4. Representative blocked-CV figures

Cohort- and fold-level blocked-CV diagnostics are shown in figures S11 and S12. The cohort residual scatter (figure S11) shows that held-out residual means remain near zero while residual spread stays below the unit-variance reference under the latent-plus-localization definition. For Biopsy 6, fold-specific paired panels show substantial train-only semivariogram variation ($\widehat{\ell}_{b,-f} = 8.6,\ 3.1,\ 4.2$ mm) and corresponding variation in held-out uncertainty reduction ($\Delta_{b,f}^{(\text{SD,test,lat})} = -0.8\%,\ 16.2\%,\ -40.9\%$), illustrating why blocked-CV summaries are aggregated over folds and biopsies rather than interpreted from one fold.

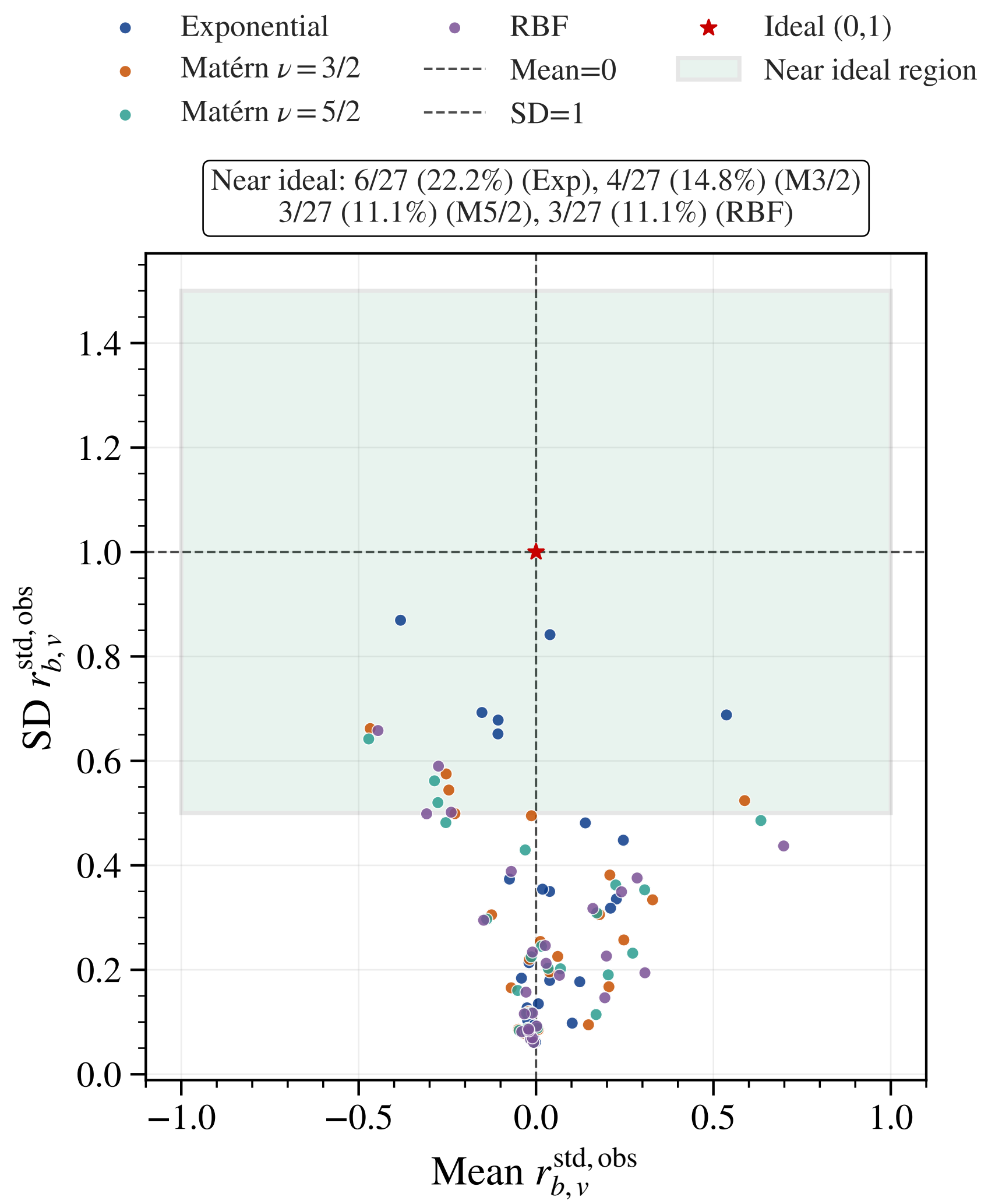

FIG. S11 Biopsy-level blocked-CV residual scatter for held-out points under the latent-plus-localization definition: mean($r_{b,v}^{\mathrm{std,obs}}$) vs SD($r_{b,v}^{\mathrm{std,obs}}$), stratified by kernel.

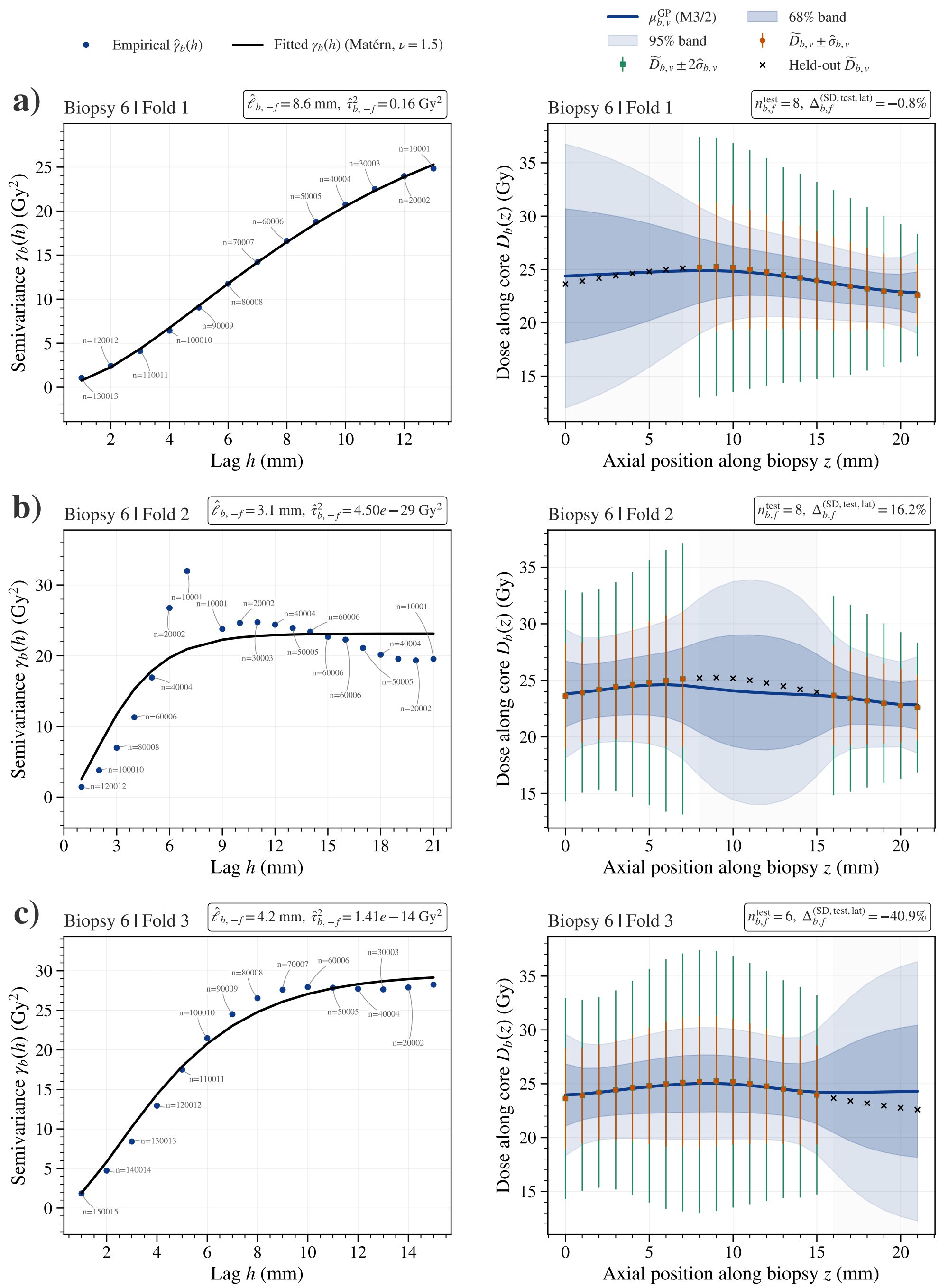

FIG. S12 Representative blocked-CV paired semivariogram-profile diagnostics for Biopsy 6 using the baseline Matérn ($\nu = 1.5$) kernel. Panels (a)–(c) show held-out folds 1–3, respectively. In each row, the left panel shows the train-only semivariogram fit and the right panel shows the corresponding profile prediction with held-out observations.

REFERENCES

[1]M. Muscat, J. Crook, A. Jirasek, J. Andrews, and N. Becker, "Dosimetric analysis along core-needle biopsies in prostate HDR brachytherapy," *Phys. Med. Biol.*, 2026.

[2]M. Muscat, J. Crook, A. Jirasek, J. Andrews, and N. Becker, "Probabilistic quality assurance of biopsy DVH thresholds in HDR prostate brachytherapy under localization uncertainty," *Journal of Applied Clinical Medical Physics*, vol. 27, no. 8, p. e70660, 2026. _eprint: https://aapm.onlinelibrary.wiley.com/doi/pdf/10.1002/acm2.70660.

[3]C. E. Rasmussen and C. K. I. Williams, *Gaussian processes for machine learning.* Adaptive computation and machine learning, Cambridge, Mass.: MIT Press, 3. print ed., 2008.

[4]M. L. Stein, *Interpolation of Spatial Data: Some Theory for Kriging.* Springer Science & Business Media, June 1999. Google-Books-ID: 5n_XuL2Wx1EC.

[5]F. Lindgren, H. Rue, and J. Lindström, "An explicit link between Gaussian fields and Gaussian Markov random fields: the stochastic partial differential equation approach," *Journal of the Royal Statistical Society: Series B (Statistical Methodology)*, vol. 73, no. 4, pp. 423–498, 2011. _eprint: https://rss.onlinelibrary.wiley.com/doi/pdf/10.1111/j.1467-9868.2011.00777.x.
